# ﻿A review of the genus *Laeocathaica* Möllendorff, 1899 (Gastropoda, Pulmonata, Camaenidae)

**DOI:** 10.3897/zookeys.1086.77408

**Published:** 2022-02-15

**Authors:** Barna Páll-Gergely, András Hunyadi, Kaibaryer Meng, Judit Fekete

**Affiliations:** 1 Centre for Agricultural Research, Plant Protection Institute, Eötvös Loránd Research Network, Herman Ottó út 15, H-1022, Budapest, Hungary Plant Protection Institute, Eötvös Loránd Research Network Budapest Hungary; 2 Adria sétány 10G 2/5., Budapest 1148, Hungary Unaffiliated Budapest Hungary; 3 National Zoological Museum of China, Institute of Zoology, Chinese Academy of Sciences, Beijing, China Institute of Zoology, Chinese Academy of Sciences Beijing China; 4 University of Pannonia, Centre of Natural Science, Research Group of Limnology, H-8200 Veszprém, Egyetem u. 10, Hungary University of Pannonia Veszprém Hungary; 5 Centre for Ecological Research, Institute of Aquatic Ecology, Department of Tisza Research, 18/c Bem square, H-4026 Debrecen, Hungary Centre for Ecological Research, Institute of Aquatic Ecology Debrecen Hungary

**Keywords:** Intraspecific variability, shell, systematics, taxonomy

## Abstract

In this paper an overview of the *Laeocathaica* species is provided, and the intraspecific variability of several *Laeocathaica* species demonstrated on multiple shells. *Laeocathaicahisanoi* Páll-Gergely, **sp. nov.** and *L.minwui* Páll-Gergely, **sp. nov.** are described based on specimens found in museum collections. Five new synonyms are recognized: *L.prionotropisalbocincta* Möllendorff, 1899 is a new synonym of *L.prionotropis* Möllendorff, 1899, *L.stenochone* Möllendorff, 1899 is a new synonym of *Laeocathaicacarinifera* (H. Adams, 1870). *Laeocathaicadistinguenda* Möllendorff, 1899, *L.tropidorhaphe* Möllendorff, 1899, and *L.dangchangensis* Chen & Zhang, 2004 are moved to the synonymy of *Laeocathaicaamdoana* Möllendorff, 1899. Furthermore, photos of paratypes of *Cathaicabizonalis* Chen & Zhang, 2004 are published for the first time.

## ﻿Introduction

The genus *Laeocathaica* Möllendorff, 1899 consists of approximately 20 species, and inhabits west China. Most of the species assigned to this genus were reported from the southern part of Gansu Province and the neighbouring Sichuan. A single species, *L.filippina* (Heude, 1882) is known from Hubei Province, more than 500 km southeast from southern Gansu.

The monophyly of this genus is questionable for several reasons. First, *Laeocathaica* is defined by the sinistral shell coiling, whereas species with dextral shells of otherwise similar appearance (large, depressed shells with white base colour and brownish spiral bands) are included in other genera such as *Cathaica* Möllendorff, 1884, *Bradybaena* Beck, 1837, and *Euhadra* Pilsbry, 1890 (Chen and Zhang 2004). The type species of the latter is *Helixpeliomphala* L. Pfeiffer, 1850 from Japan, which makes it questionable that the same large-bodied land snail genus could inhabit such a vast area covering ca. 3500 km. Moreover, species similar to *Laeocathaica* species (e.g., *Bradybaenahaplozona* Möllendorff, 1899) have been classified in genera different from *Laeocathaica* (Möllendorff 1899; Chen and Zhang 2004). Second, the genus *Laeocathaica* as understood by Möllendorff (1899) and Chen and Zhang (2004) is variable in terms of shells characters. Some are large, with a white base colour, keeled or rounded body whorl, and without apertural barriers, others are keeled with apertural barriers, and some are small, transparent, and also possess apertural teeth. The genital anatomy is known in a handful of species only: *L.prionotropis* (see Chen and Zhang 2004), *L.polytyla* (see Schileyko 2004) and *L.filippina* (see Wu 2004), providing little basis of our understanding of the systematics of *Laeocathaica* and related genera.

In this paper we provide an overview of the genus *Laeocathaica* after consulting all available types and newly collected samples. We provide precise localities for most species, and photographs of multiple shells showing intraspecific variability for the first time in this genus.

## ﻿Materials and methods

We counted the whorls of adult shells according to Kerney and Cameron (1979). Of the newly collected specimens and the ones deposited in the Senckenberg Museum, 10–20 photographs were taken of each shell using Canon EOS 6D camera and a Canon Macro Lens EF 100 mm 1: 2.8 and merged to create a single image using Photoshop. Shells deposited in other museums were photographed by the respective museum staff.

### ﻿Abbreviations

**D** shell diameter;

**H** shell height.

### ﻿Depositories

**ANSP**Academy of Natural Sciences (Philadelphia, USA);

**HA** Collection András Hunyadi (Budapest, Hungary);

**IZCAS**National Zoological Museum of China, Institute of Zoology, Chinese Academy of Sciences (Beijing, China);

**MNHN** Muséum National d’Histoire Naturelle (Paris, France);

**NHM** The Natural History Museum (London, UK);

**NHMUK** When citing lots deposited in the NHM;

**PGB** Collection Barna Páll-Gergely (Budapest, Hungary);

**SMF**Senckenberg Forschungsinstitut und Naturmuseum (Frankfurt am Main, Germany);

**USNM**Smithsonian National Museum of Natural History (Washington, USA).

## ﻿Taxonomy and systematics

### ﻿Family Camaenidae Pilsbry, 1895

#### ﻿Genus Cathaica

Taxon classificationAnimaliaStylommatophoraBradybaenidae

Möllendorff, 1884

9FADF5B3-B862-52A8-AD82-ECBA5472DA80

##### Type species.

*Helixpyrrhozona* Philippi, 1845; OD.

#### ﻿ Cathaica (Cathaica) bizonalis

Taxon classificationAnimaliaStylommatophoraBradybaenidae

Chen & Zhang, 2004

0E019B5F-8B22-53E6-A3EB-E1D1F749A548

Figure 1

Cathaica (Cathaica) bizonalis Chen & Zhang, 2004: 238 [Chinese description], fig. 219 (erroneous figure showing L.carinalis specimen); 439 [English description].

##### Type locality.

陕西洛川县黑木沟. “Hemugou twon [sic!], Luochuan County (35°7'N, 109°04'E), Shaanxi Province”.

##### Remarks.

Chen and Zhang (2004) described two species relevant for the present study: Cathaica (Cathaica) bizonalis Chen & Zhang, 2004 and *Laeocathaicacarinalis* Chen & Zhang, 2004. According to the original description of *Cathaicabizonalis* (page 238 for Chinese, page 439 for English description), it is a dextral species characterised by a keel and two spiral bands (one above and one below the keel), and it is known from Shaanxi Province. The accompanying figure (Chen and Zhang 2004: fig. 219), however, shows a sinistral *Laeocathaica* species, identified here as *L.carinalis*.

According to the original description of *Laeocathaicacarinalis*, that species is characterised by a sinistral, strongly depressed, keeled shell with a broad umbilicus. However, the provided photo (Chen and Zhang 2004: fig. 334) shows a sinistral juvenile shell of probably a *Laeocathaica* species with blunt keel and narrow umbilicus.

**Figure 1.**
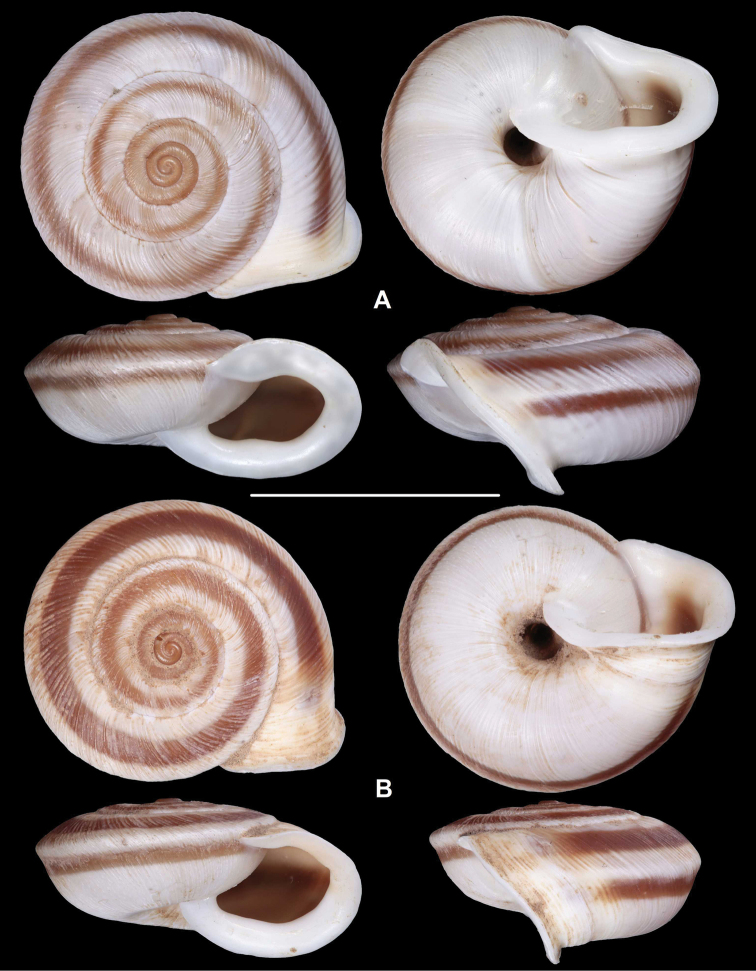
Paratypes of Cathaica (Cathaica) bizonalis Chen & Zhang, 2004 **A**IZCAS TM 097593 **B**IZCAS TM 097600. Scale bar: 10 mm. Photographs: Kaibaryer Meng.

**Figure 2.**
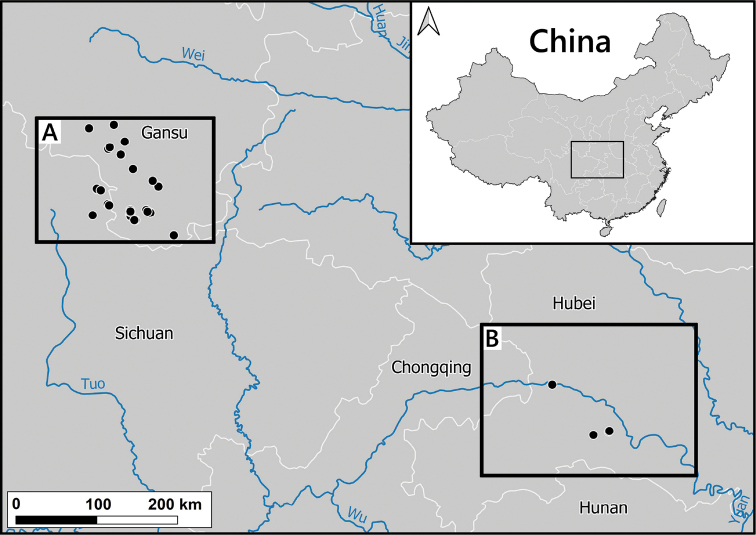
Distribution of the genus *Laeocathaica* in China **A** see Figs 8, 11, 17, 24 for details **B** see Fig. 14 for detail.

Examining the specimens deposited in IZCAS revealed the following:

1. The shell labelled as the holotype of Cathaica bizonalis is a dextral, juvenile shell.
2. A jar labelled as paratypes of Cathaica bizonalis (IZCAS TM 006579–006595) contains several probably conspecific juvenile specimens in ethanol.
3. Another jar labelled as paratypes of Cathaica bizonalis (IZCAS TM 097575–097587) contained 13 dry adult shells of a Laeocathaica carinalis (sinistral, strongly keeled species).
4. Among those shells (IZCAS TM 097575–097587), specimen IZCAS TM 097578 is exactly the same shell as in fig. 219 of Chen and Zhang (2004).
5. A jar labelled as paratypes of Laeocathaica carinalis (IZCAS TM 006612–006625) contained several adult specimens of L. carinalis in ethanol.
6. Another jar labelled as paratypes of Laeocathaica carinalis (IZCAS TM 097588–097602) contained several dry adult shells of Cathaica bizonalis (two of them figured in this work, see Fig. 1). This clearly shows that shells of the above taxa have been confused even before the publication. As a result, not only were the different samples mixed up with the labels in the collection, but also in the original descriptions.

Based on Chen and Zhang (2004) and the examination of the specimens deposited in IZCAS, we conclude the following:

1. Fig. 219 in Chen and Zhang (2004) shows the holotype of Laeocathaica carinalis Chen & Zhang, 2004 instead of Cathaica bizonalis.
2. Fig. 334 in Chen and Zhang (2004) shows an unidentifiable juvenile Laeocathaica species, and not Laeocathaica carinalis.
3. A holotype was designated in the original description of Cathaica bizonalis, so a lectotype cannot be chosen later. The holotype cannot be located because there is no photograph in the original description, and because the paratypes are of similar size (i.e., the given measurements of the holotype are insufficient to recognize the shell). So, we treat it as lost (perhaps in the lot of paratypes). Since all the paratypes belong to one single species, and are consistent with the original description, we know what the authors intended by the taxon, so designation of a neotype is not needed.

#### ﻿Genus Laeocathaica

Taxon classificationAnimaliaStylommatophoraBradybaenidae

Möllendorff, 1899

02F1D21C-0BA9-515F-A78F-ED542813E631

Laeocathaica Möllendorff, 1899: 86; Richardson 1983: 77; Schileyko 2004: 1686; Chen and Zhang 2004: 312.

##### Type species.

Helix (Plectotropis) christinae H. Adams, 1870 (by original designation).

#### ﻿ Laeocathaica amdoana

Taxon classificationAnimaliaStylommatophoraBradybaenidae

Möllendorff, 1899

4CBFCF0D-A923-55C8-80AD-F3DEEF019CB6

**Figure 3.**
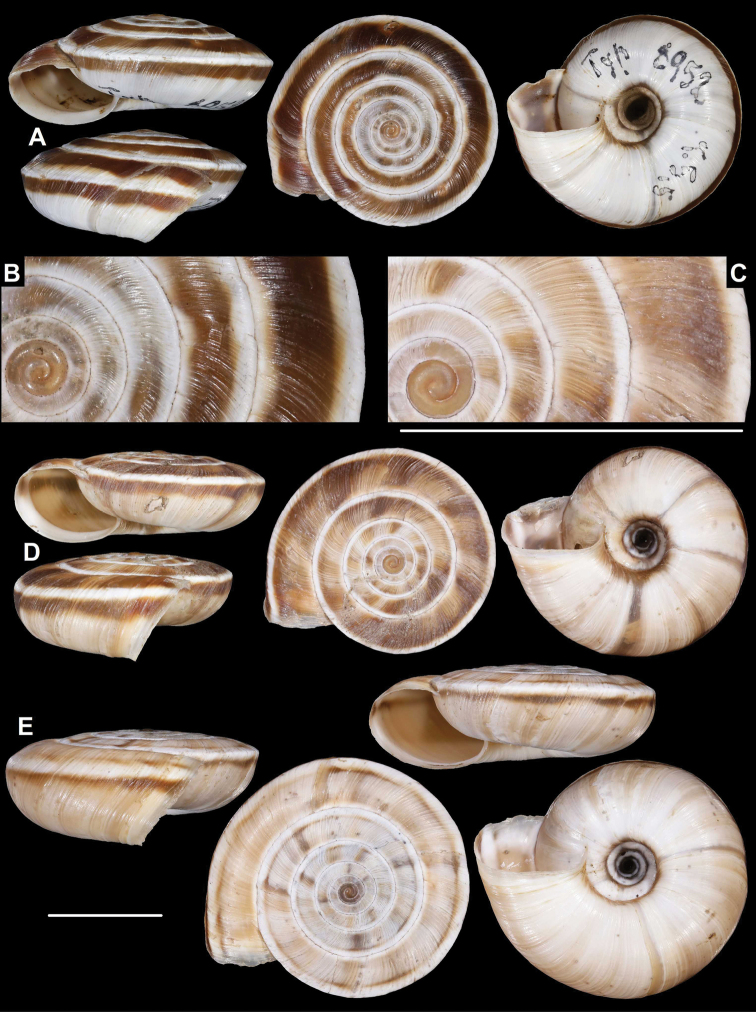
*Laeocathaicaamdoana***A, B** lectotype (SMF 8952) **C, D** 2016/74, specimen2 **E** 2016/74, specimen1. Scale bars: 10 mm. All photographs: B. Páll-Gergely.

**Figure 4.**
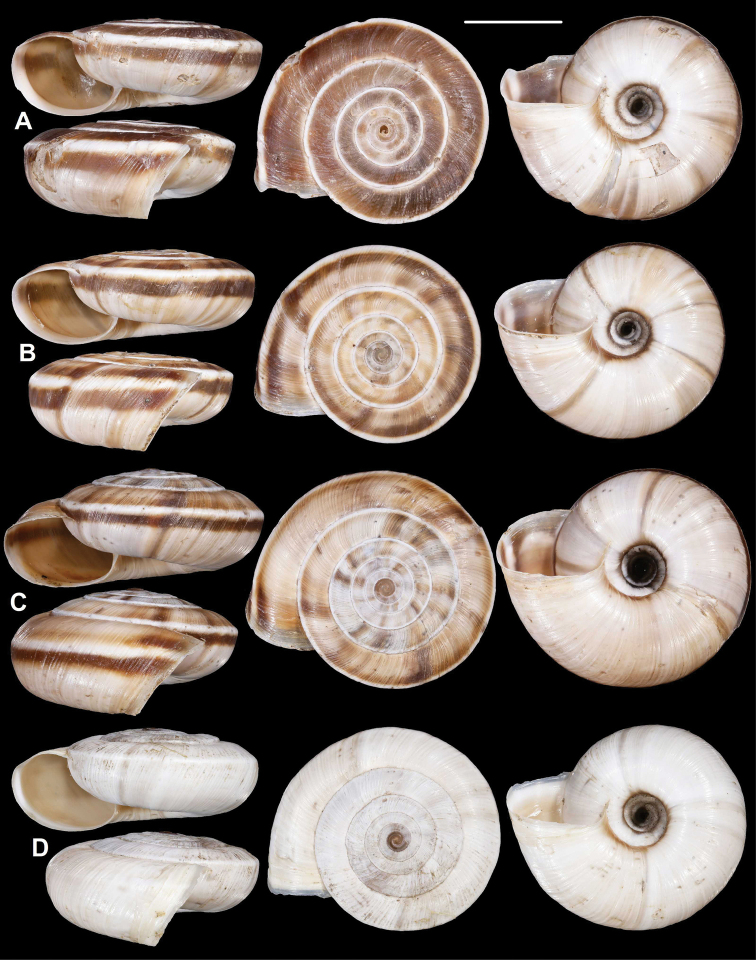
*Laeocathaicaamdoana* Möllendorff, 1899, 2016/75 (4 different shells of the same sample). Scale bar: 10 mm. All photographs: B. Páll-Gergely.

**Figure 5.**
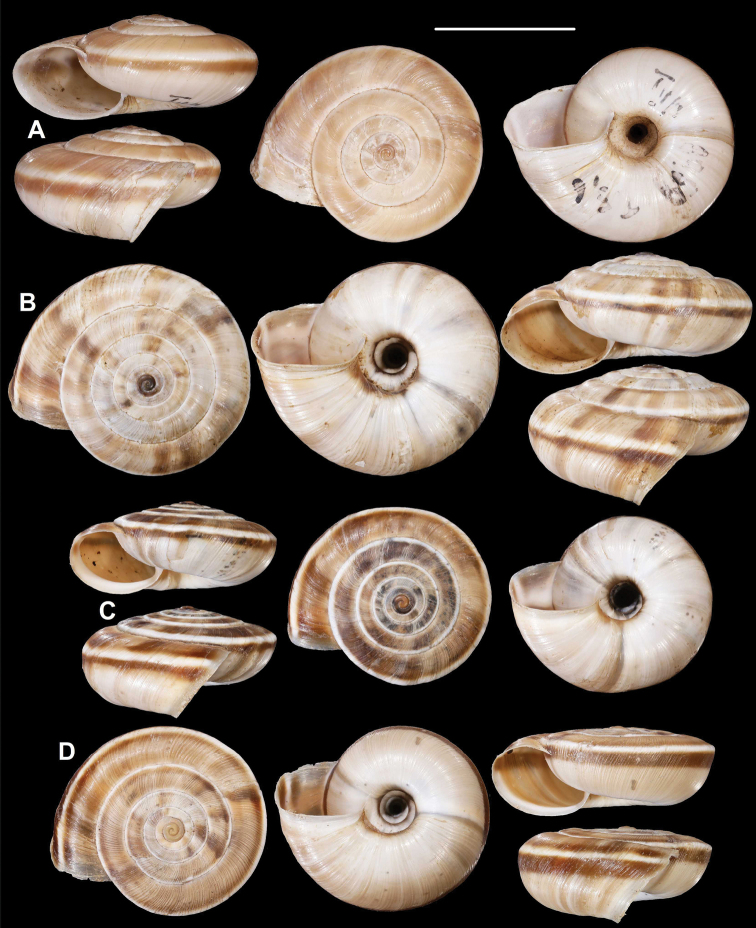
*Laeocathaicaamdoana* Möllendorff, 1899 **A** lectotype of *L.distinguenda* (SMF 8959) **B** 2016/72, specimen1 **C** 2016/72, specimen2 **D** 2016/76. Scale bar: 10 mm. All photographs: B. Páll-Gergely.

**Figure 6.**
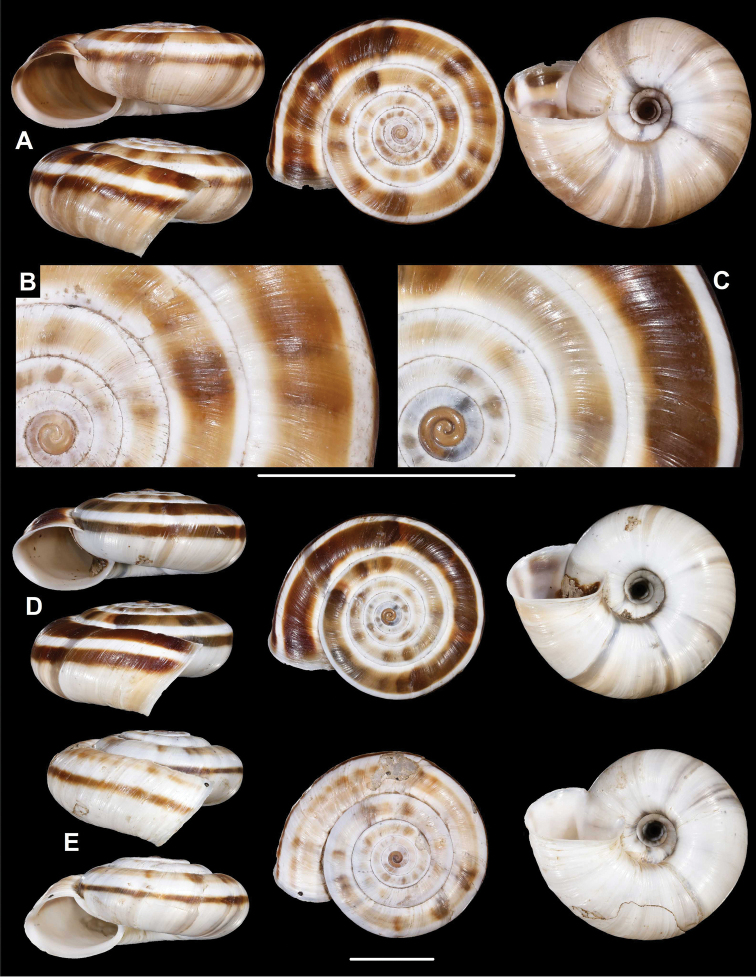
*Laeocathaicaamdoana* Möllendorff, 1899 **A, B** 2016/70a **C, D** 2016/71 **E** 2016/69. Scale bars: 10 mm. All photographs: B. Páll-Gergely.

**Figure 7.**
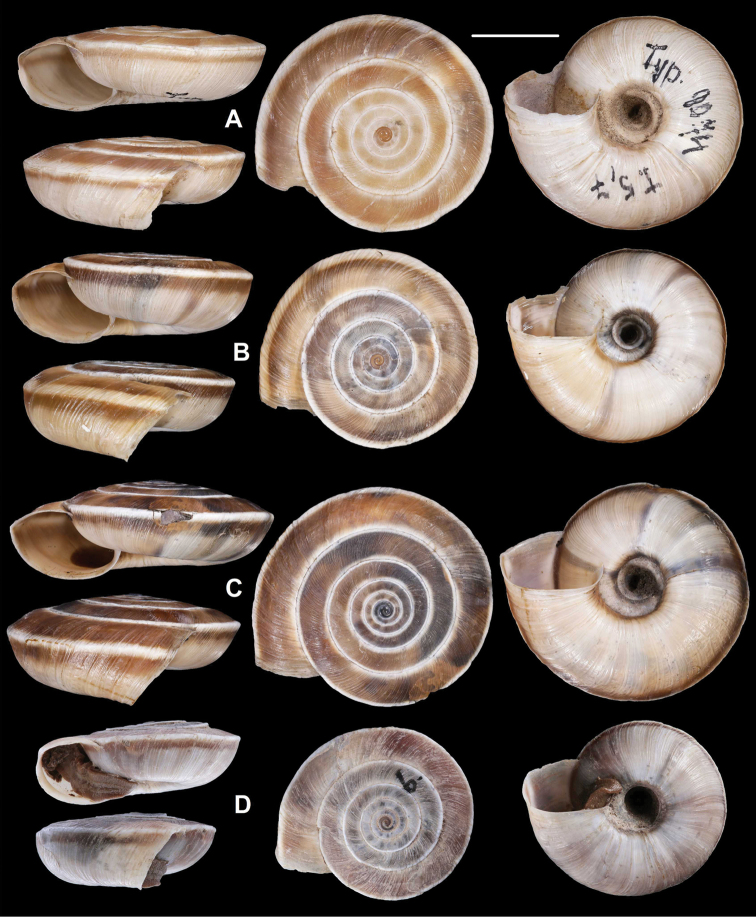
*Laeocathaicaamdoana* Möllendorff, 1899 **A** lectotype of *Laeocathaicatropidorhaphe* (SMF 9074) **B** 2016/88 **C** 2016/89 **D** paratype of *Laeocathaicadangchangensis* Chen & Zhang, 2004 (labelled as holotype). Scale bar: 10 mm. Photographs: K. Meng (**D**) and B. Páll-Gergely (**A–C**).

Figures 3 , 4 , 5 , 6 , 7

Laeocathaica amdoana Möllendorff, 1899: 92–93, pl. 5, fig. 5.
Laeocathaica distinguenda Möllendorff, 1899: 93, pl. 5, fig. 6. **new synonym**
Laeocathaica tropidorhaphe Möllendorff, 1899: 94, pl. 5, fig. 7. **new synonym**
Laeocathaica amdoana . – Yen 1939: 148, pl. 15, fig. 31.
Laeocathaica distinguenda . – Yen 1939: 149, pl. 15, fig. 32.
Laeocathaica tropidorhaphe . – Yen 1939: 149, pl. 15, fig. 33.
Laeocathaica (Laeocathaica) amdoana . – Zilch 1968: 173.
Laeocathaica (Laeocathaica) distinguenda . – Zilch 1968: 173.
Laeocathaica (Laeocathaica) tropidorhaphe . – Zilch 1968: 175.
Laeocathaica amdoana . – Chen & Zhang 2004: 316, fig. 303.
Laeocathaica distinguenda . – Chen & Zhang, 2004: 318, fig. 305.
Laeocathaica tropidorhaphe . – Chen & Zhang 2004: 319, fig. 307.
Laeocathaica dangchangensis Chen & Zhang, 2004: 339 [Chinese description], 443 [English description], fig. 332. **new synonym**

##### Type material.

China (Gansu), Ho-dshi-gou, coll. Möllendorff ex coll. Potanin 853, SMF 8952 (lectotype of *amdoana*, Fig. 3A, B) • China (SO-Gansu), Wen-Hsien, coll. Möllendorff ex coll. Potanin 907, SMF 8953 (paralectotype of *amdoana*) • China (Sy-tshuan): Thal des Pui-hob. Lum-du, coll. Möllendorff ex coll. Potanin 906, SMF 8959 (lectotype of *distinguenda*, Fig. 5A) • SO-Gansu (NW China), coll. C.R. Boettger ex coll. Möllendorff, SMF 95024/1 paralectotype of *distinguenda* • SO-Gansu: Nanping, coll. Möllendorff ex coll. Potanin 8, 64, 846, SMF 8958/5 paratypes of *distinguenda* • SO-Gansu, Yü-Lin-Guam, u. Wen-hsien, coll. Möllendorff ex coll. Potanin 11, 521, 565, SMF 8955/3 paralectotypes of *distinguenda* • China: SO-Gansu, coll. Möllendorff ex coll. Potanin 725a, SMF 8957/3 paralectotypes of *distinguenda* • Shy-Pu am Pui-hu, coll. Möllendorff ex coll. Potanin 69, 653, SMF 8956/2 paralectotypes of *distinguenda* • SO-Gansu, zw. Li-dshia-pu u. Hsi-gu-tsheng, coll. Möllendorff ex coll. Potanin 923, SMF 9074 (lectotype of *L.tropidorhaphe*, Fig. 7A) • Same data, SMF 9077/2 paralectotypes • NW-China, SO-Gansu, coll. C.R. Boettger 1904 ex coll. Möllendorff SMF 95126/1 (paralectotype of *L.tropidorhaphe*) • SO-Gansu, Tan-tshang, coll. Möllendorff ex coll. Potanin 545, 623, 808, SMF 9075/4 (paralectotypes of *L.tropidorhaphe*) • Gansu: Dshie-dshou, coll. Möllendorff ex coll. Potanin 119, SMF 9076/1 (paralectotype of *L.tropidorhaphe*) • IZCAS TM 095895 (labelled as holotype of *L.dangchangensis*, but its measurements do not match with the ones given in the original description, see also remarks, Fig. 7D) • IZCAS TM 095843–095894 + IZCAS TM 095896–095950 (103 paratypes of *L.dangchangensis* in ethanol).

##### Museum material.

Szechwan, coll. Möllendorff, SMF 42564/1 • Tan-Tschan, coll. Möllendorff, SMF 24269/1 (“*tropidorhape*”).

##### New material (typical tropidorhaphe).

**Figure 8.**
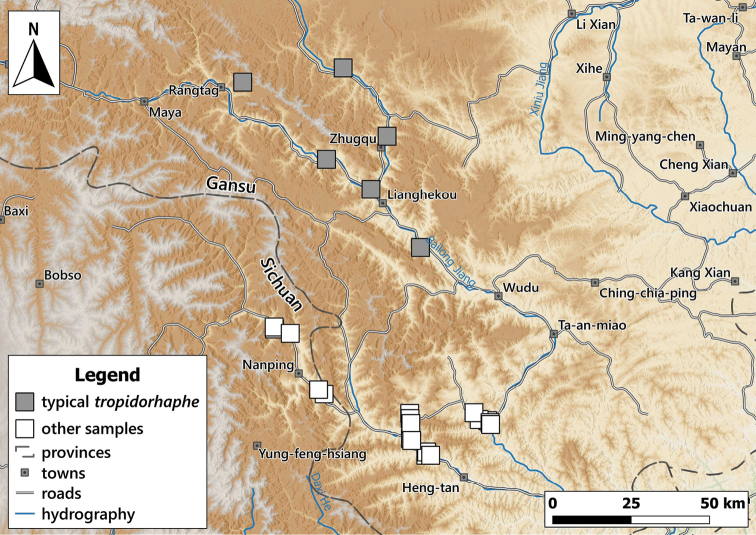
Distribution of *Laeocathaicaamdoana* Möllendorff, 1899 in China (detail from Fig. 2A).

China • 2 shells; Gansu, Longnan Shi, Dangchang Xian, Chengguan Zhen, Wenchangmiao, temple hill (locality code: 2016/87); 34°02.394'N, 104°23.499'E; 1835 m a.s.l.; 02 June 2016; A. Hunyadi leg.; HA • 2 shells; Gansu, Longnan Shi, Dangchang Xian, Lianghekou Xiang, Lianghekou Cun, rock above the intersection (locality code: 2016/89); 33°41.808'N, 104°29.182'E; 1245 m a.s.l.; 02 June 2016; A. Hunyadi leg.; HA (Fig. 8C) • 2 shells; Gansu, Longnan Shi, Dangchang Xian, Guanting Zhen, 1.5 km north of Guanting towards Dangchang (locality code: 2016/88); 33°50.803'N, 104°32.470'E; 1815 m a.s.l.; 02 June 2016; A. Hunyadi leg.; HA (Fig. 7B) • 2 shells; Gansu, Longnan Shi, Zhugqu Xian, 2.5 km west of Suoertou Cun, northern bank of Bailong He (locality code: 2016/91); 33°46.906'N, 104°20.106'E; 1235 m a.s.l.; 03 June 2016; A. Hunyadi leg.; HA • 2 shells; Gansu, Longnan Shi, Wudu Xian, Jiaogong Zhen, 1.5 km west of Chenjiaba Cun, Zhaoyangdong, below the cave (locality code: 2016/95); 33°31.924'N, 104°39.286'E; 1175 m a.s.l.; 04 June 2016; A. Hunyadi leg.; HA.

##### New material (other morphs).

6 shells; Sichuan, Aba, Jiuzhaigou Xian, Baihe Xiang, southern edge of Taiping Cun, rock wall facing north (locality code: 2016/72); 33°18.026'N, 104°09.500'E; 30 May 2016; A. Hunyadi leg.; HA (Fig. 5B, C) • 1 shell; Sichuan, Aba, Jiuzhaigou Xian, Baihe Xiang, Taiping Cun, eastern bank of Baishui He (locality code: 2016/73); 33°18.366'N, 104°09.413'E; 30 May 2016; A. Hunyadi leg.; HA • 4 shells; Sichuan, Aba, Jiuzhaigou Xian, Anle Xiang, ca. 1.5 km east of Zhongtianshan Cun towards Jiuzhaigou Shi (locality code: 2016/74); 33°17.279'N, 104°12.702'E; 1445 m a.s.l.; 30 May 2016; A. Hunyadi leg.; HA (Fig. 3C–E) • 4 shells; Sichuan, Aba, Jiuzhaigou Xian, Guoyuan Xiang, 1 km from Guoyuanyi Cun towards Lengshuishan Cun (locality code: 2016/75); 33°07.681'N, 104°18.489'E; 1220 m a.s.l.; 30 May 2016; A. Hunyadi leg.; HA (Fig. 4) • 1 shell; Sichuan, Aba, Jiuzhaigou Xian, Guoyuan Xiang, Guoyuaner Cun, environment of the bridge (locality code: 2016/76); 33°06.922'N, 104°19.617'E; 1200 m a.s.l.; 30 May 2016; A. Hunyadi leg.; HA (Fig. 5D) • 3 shells; Gansu, Longnan Shi, Wenxian, Buziba Xiang, southern edge of Buziba Cun, western bank of the river (locality code: 2016/77); 33°03.592'N, 104°37.094'E; 1215 m a.s.l.; 31 May 2016; A. Hunyadi leg.; HA • 1 shell; Gansu, Longnan Shi, Wenxian, Buziba Xiang, northern edge of Taojiaba Cun, 200 m towards Buziba (locality code: 2016/78); 33°02.706'N, 104°37.157'E; 1200 m a.s.l.; 31 May 2016; A. Hunyadi leg.; HA • 4 shells; Gansu, Longnan Shi, Wenxian, Buziba Xiang, 1 km south of Taojiaba Cun towards Dongyukou Cun (locality code: 2016/79); 33°01.865'N, 104°37.329'E; 1150 m a.s.l.; 31 May 2016; A. Hunyadi leg.; HA • 4 shells; Gansu, Longnan Shi, Wenxian, Chengguan Zhen, next to a museum (locality code: 2016/64); 32°56.471'N, 104°40.379'E; 960–970 m a.s.l.; 28 May 2016; A. Hunyadi leg.; HA • 3 shells; Gansu, Longnan Shi, Wenxian, Chengguan Zhen, cemetery hill above the city (locality code: 2016/65); 32°57.026'N, 104°40.527'E; 1090 m a.s.l.; 28 May 2016; A. Hunyadi leg.; HA • 4 shells; Gansu, Longnan Shi, Wenxian, Jianshan Xiang, 1200 m south of Hekou Cun, eastern bank of Bailong He (locality code: 2016/68); 33°01.703'N, 104°53.602'E; 810 m a.s.l.; 29 May 2016; A. Hunyadi leg.; HA • 2 shells; Gansu, Longnan Shi, eastern edge of Wenxian, northern bank of the river (locality code: 2016/66); 32°56.459'N, 104°41.372'E; 28 May 2016; A. Hunyadi leg.; HA • 4 shells; Gansu, Longnan Shi, Wenxian, Jianshan Xiang, southern edge of Hekou Cun, western bank of Bailong He (locality code: 2016/67); 33°02.014'N, 104°53.478'E; 800 m a.s.l.; 29 May 2016; A. Hunyadi leg.; HA • 3 shells; Gansu, Longnan Shi, Wenxian, Shifang Xiang, 800 m from the northwestern edge of Baiyiba Cun towards Dongyukou Cun, left bank of the river (locality code: 2016/83); 32°58.985'N, 104°37.503'E; 970 m a.s.l.; 31 May 2016; A. Hunyadi leg.; HA • 4 shells; Gansu, Longnan Shi, Wenxian, Shifang Xiang, 1300 m northwest from Baiyiba Cun towards Dongyukou Cun (locality code: 2016/82); 32°59.346'N, 104°37.233'E; 980 m a.s.l.; 31 May 2016; A. Hunyadi leg.; HA • 9 shells; Gansu, Longnan Shi, Wenxian, Jianshan Xiang, western edge of Hekou Cun towards Caojiaba, along road no. 212 (locality code: 2016/69); 33°2.343'N, 104°53.045'E; 29 May 2016; A. Hunyadi leg.; HA (Fig. 6E) • 8 shells; Gansu, Longnan Shi, Wenxian, Jianshan Xiang, 600 m west of Jianshan towards Diaohuya (locality code: 2016/70a); 33°02.559'N, 104°51.254'E; 850 m a.s.l.; 29 May 2016; A. Hunyadi leg.; HA (Fig. 6A, B) • 4 shells; Gansu, Longnan Shi, Wenxian, Jianshan Xiang, 200 m north of Lvjiaba, along road no. 212 (locality code: 2016/71); 33°03.712'N, 104°50.209'E; 29 May 2016; A. Hunyadi leg.; HA (Fig. 6C, D).

##### Distribution.

This species is known from several sites in southwestern Gansu Province and the neighbouring areas in Sichuan (Fig. 8).

##### Remarks.

*Laeocathaicaamdoana* was described from Pass Ho-Dshi-Gou bei Mu-gua-tshi (the exact locality could not be located on the map) near Wenxian at the border with Sichuan province, and characterised by a domed, brown, dorsal side with a relatively broad, sharp, distinctly bordering, white band of the keel. We did not find shells identical to typical *Laeocathaicaamdoana*, but have found similar ones that can be identified as conspecific (Figs 3C, D, 4).

*Laeocathaicadistinguenda* is represented in the Senckenberg Museum by several samples. Strangely, the lectotype is the most “untypical” among the lots labelled as *L.distinguenda* due to its pale caramel colour, the blurry border of light and dark stripes, and the rounded body whorl. Our samples from the vicinity of Wenxian (Fig. 5B–D) are more similar to the paralectotypes of *L.distinguenda*.

*Laeocathaicatropidorhaphe* was described from the north (Tanchang and its vicinity), and is characterised by a large, keeled shell with a flat dorsal side and a thick brown spiral band. The northernmost populations we collected (samples 2016/87, 2016/88, 2016/89, 2016/91, 2016/95) agree with the types of *L.tropidorhaphe* in size, shape, and colouration, but their spire height is variable. However, some shells from much further south are also similar (i.e., samples 2016/74, 2016/75, see Fig. 4). The characteristic colouration of *L.tropidorhaphe* (brown dorsal side with slender white band on the keel) is also not unique to the northern *L.tropidorhaphe* populations, but can be found in more southern populations as well (compare Fig. 4a with Fig. 7).

Overall, there is a continuous variation across most of the historical and newly collected samples in terms of shape of dorsal side, shape of body whorl, size, colour, and sculpture (see Figs 3B, C; 6B, C). *Laeocathaicaamdoana* and the lectotype of *L.distinguenda* (but not the paralectotypes!) seem to be slightly out of the morphological continuum, but not to a degree that a species-level distinction should be applied.

Colouration can be extremely variable even within a single population (see Fig. 4). Therefore, colour is of minor importance in species distinction within this group of *Laeocathaica*. It is a general trend that towards the southeastern part of the distribution of these “species”, the peripheral keel disappears and the body whorl becomes rounded.

Because of the aforementioned reasons, and until anatomical and DNA sequence data become available, we do not find the names *L.distinguenda* and *L.tropidorhaphe* meaningful, and so we provisionally synonymise them with *L.amdoana*. Table 1 summarises the key traits that are variable across and within newly collected populations.

*Laeocathaicadangchangensis* Chen & Zhang, 2004 is also a junior synonym of *L.amdoana*, because it shows the same characteristic conchological features (large shell size, acute, white keel, almost flat dorsal side) as *L.tropidorhaphe*. Moreover, its type locality (Shawan town, Dangshang county (34°0'N, 104°3'E), Gansu Province, China) is situated close to the known sites of *L.tropidorhaphe*, whose two closest populations are situated at ca. 31 km and 35 km from the type locality of *L.dangchangensis*.

According to the original description of *Laeocathaicadangchangensis*, the holotype has a shell width of 27.22 mm. However, the shell labelled as the holotype is 23 mm wide. Moreover, the number 6 is written on that specimen’s dorsal side, whereas “Holotype: Sp8” is written on the label. We have not found any shells bearing the number 8. Consequently, the shell labelled as the holotype is a paratype, and the real holotype is probably one of the specimens labelled as paratypes, or lost.

#### ﻿ Laeocathaica carinalis

Taxon classificationAnimaliaStylommatophoraBradybaenidae

Chen & Zhang, 2004

ACC601C8-7474-5647-AAF5-598883342EE4

Figures 9 , 10

Laeocathaica carinalis Chen & Zhang, 2004: 341 [Chinese description], fig. 334 (erroneous! Shows a juvenile Laeocathaica shell belonging to another species); 444 [English description].

##### Type locality.

“Town of Wenxian County, (33°0'N, 104°6'E), Gansu Province, China”.

##### Type material.

**Figure 9.**
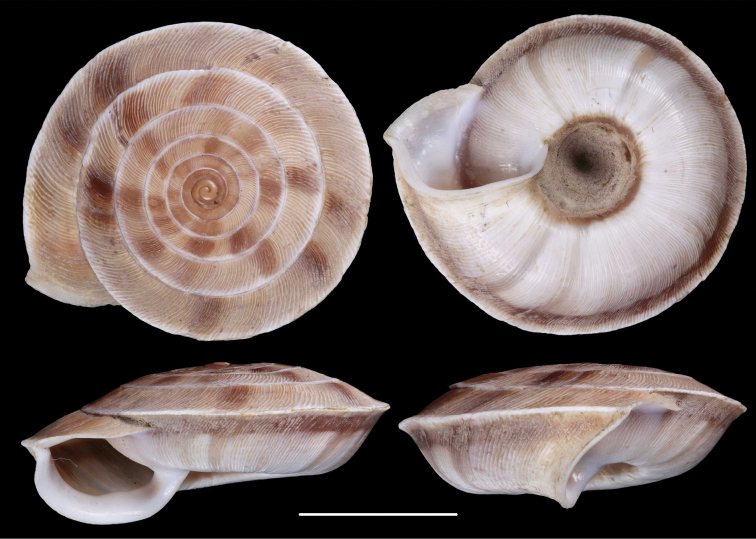
*Laeocathaicacarinalis* Chen & Zhang, 2004 (IZCAS TM 097578, holotype). Scale bar: 10 mm. Photographs: Kaibaryer Meng.

The shell we examined (IZCAS TM 097578) is exactly the same as the one figured by Chen and Zhang (2004: fig. 219) as the holotype of *Cathaicabizonalis* Chen & Zhang, 2004. Therefore, we understand this situation as a confusion of specimens and photographs before publication, and consider the figured shell (IZCAS TM 097578, Fig. 9) as the holotype. See also under *Cathaicabizonalis* Chen & Zhang, 2004.

##### New material.

**Figure 10.**
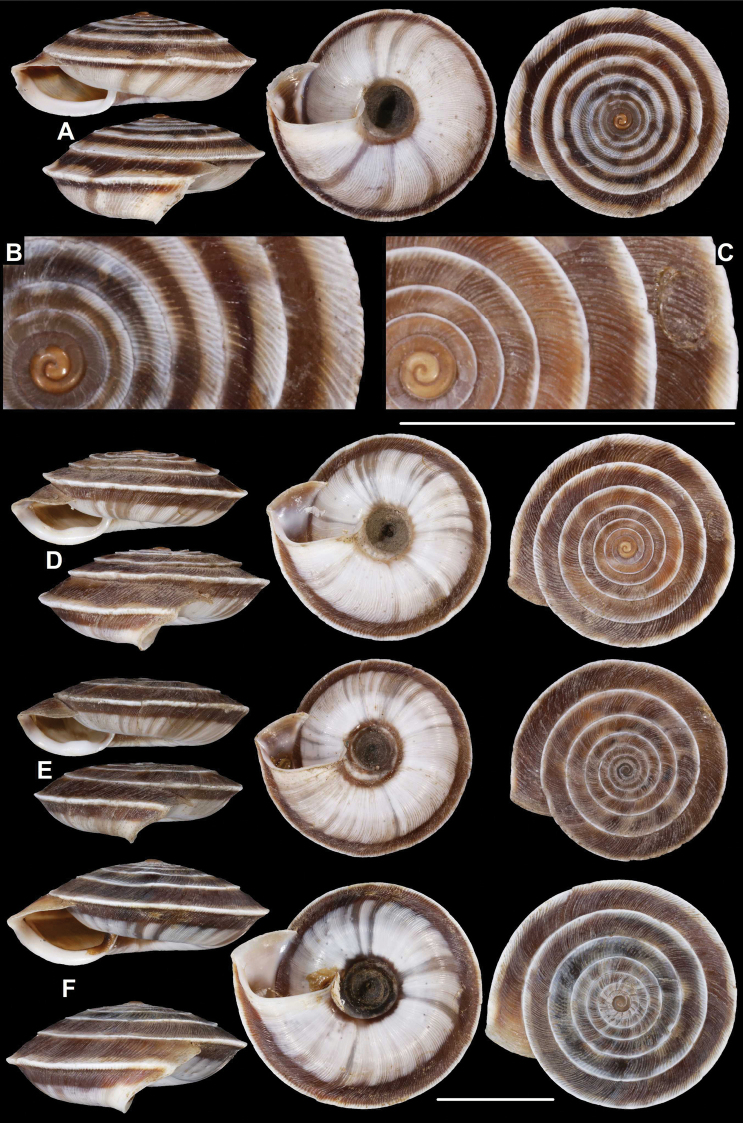
*Laeocathaicacarinalis* Chen & Zhang, 2004 **A, B** 2016/70b **C, D** 2016/81 **E** 2016/82 **F** 2016/83. Scale bars: 10 mm. All photographs: B. Páll-Gergely.

China • 4 shells; Gansu, Longnan Shi, Wenxian, Jianshan Xiang, 1800 m west of Jianshan towards Diaohuya, right side of road no. 212 (locality code: 2016/70b); 33°2.922'N, 104°50.840'E; 29 May 2016; A. Hunyadi leg.; HA (Fig. 10A, B) • 10 shells; Gansu, Longnan Shi, Wenxian, Shifang Xiang, 1300 m northwest from Baiyiba Cun towards Dongyukou Cun, right side of the road (locality code: 2016/82); 32°59.346'N, 104°37.233'E; 980 m a.s.l.; 31 May 2016; A. Hunyadi leg.; HA (Fig. 10E) • 4 shells; Gansu, Longnan Shi, Wenxian, Shifang Xiang, 800 m from the northwestern edge of Baiyiba Cun towards Dongyukou Cun, left bank of the river (locality code: 2016/83); 32°58.985'N, 104°37.503'E; 970 m a.s.l.; 31 May 2016; A. Hunyadi leg.; HA (Fig. 10F) • 7 shells; Gansu, Longnan Shi, Wenxian, Buziba Xiang, 1 km south of Taojiaba Cun towards Dongyukou Cun (locality code: 2016/79); 33°01.865'N, 104°37.329'E; 1150 m a.s.l.; 31 May 2016; A. Hunyadi leg.; HA • 5 shells; Gansu, Longnan Shi, Wenxian, Buziba Xiang, southern edge of Liangjiaba (locality code: 2016/81); 33°00.262'N, 104°36.712'E; 1005 m a.s.l.; 31 May 2016; A. Hunyadi leg.; HA (Fig. 10C, D) • 1 shell; Gansu, Longnan Shi, Wenxian, Chengguan Zhen, next to a museum (locality code: 2016/64); 32°56.471'N, 104°40.379'E; 960–970 m a.s.l.; 28 May 2016; A. Hunyadi leg.; HA.

**Table 1.** Shell morphological traits of *Laeocathaicaamdoana* Möllendorff, 1899 populations.

Locality no.	D (in mm)	H (in mm)	Dorsal side	Body whorl
2016/64	21.9–27.1	12–14.7	domed	rounded to slightly keeled
2016/65	25.5–26.2	13.3–14.7	domed	rounded to slightly keeled
2016/66	23.7–24.5	11.2–12.9	domed	rounded
2016/67	19.9–23.3	8.8–11	slightly domed	rounded
2016/68	24.3–25.4	10.4–10.7	flat to slightly domed	rounded
2016/69	27.4–30.7	12.2–14.1	slightly domed	rounded
2016/70a	30–31.3	13.1–14.9	slightly domed	rounded
2016/71	28.5–29.7	13–13.6	slightly domed	rounded
2016/72	19.8–24.2	9.1–12.2	domed	slightly keeled
2016/73	21.8	10	domed	slightly keeled
2016/74	21.4–25.4	9–10.7	flat to slightly domed	strongly to slightly keeled
2016/75	25.1–27.4	9.8–12.5	slightly domed to domed	strongly to slightly keeled
2016/76	20.8	9	flat	strongly keeled
2016/77	20.8–21.6	10.5–11.5	domed	slightly keeled
2016/78	22.7	11.6	slightly domed	slightly keeled
2016/79	20.7–21.7	10–10.3	slightly domed to domed	strongly to slightly keeled
2016/82	20.6–23	9.8–11.6	slightly domed to domed	rounded to slightly keeled
2016/83	27.7–28.3	13–13.7	slightly domed	rounded
2016/87	23–26.9	8.1–11.4	flat to slightly domed	strongly keeled
2016/88	26.6–27.7	10.9–11.3	flat to slightly domed	strongly keeled
2016/89	27.9–30.6	11.3–12.1	flat to slightly domed	strongly keeled
2016/91	27.5–30	10.4–11.5	flat	strongly keeled
2016/95	23.6–27.4	9–10.1	flat	strongly keeled

##### Description.

Shell sinistral, depressed, strongly keeled, dorsal side with flat, scalariform whorls; ventral side widely conical; dorsal side chocolate brown, ornamented with a white keel on all whorls; ventral side primarily white, below the white keel there is a chocolate brown belt, white part ornamented with greyish radial stripes that sometimes reach the umbilicus, but sometimes thin and stop before umbilicus; umbilicus inside with a chocolate-brown and a white belt; entire shell consists of 7.25–7.75 whorls; protoconch consists of 1.5–1.75 whorls, brownish, seemingly smooth, extremely finely granulose, rather matte, slightly protruding above first whorls of teleoconch; white keels of every whorl slightly elevated from dorsal surface, but dorsal surface flat with usually the last one being scalariform; dorsal side with fine, irregular wrinkles and between the main wrinkles there are very fine radial lines; ventral surface with less prominent wrinkles; umbilicus rather narrow, funnel-shaped, shows all whorls; periumbilical keel absent; aperture oblique to shell axis, semilunar, with pointed incision at the keel; peristome expanded and slightly thickened, but not reflexed; palatal swelling whitish, with a low, blunt basal tooth; parietal callus practically absent, in some old specimens with translucent calcareous layer.

**Measurements** (in mm): D: 18.6–22.9; H: 6.8–9.8 (*n* = 13, newly collected shells from multiple samples).

##### Differential diagnosis.

The most similar species is *L.pewzowi*, which is smaller, paler in colour, has a wider umbilicus, a more domed (and not scalariform) dorsal side, stronger radial sculpture, and a more oblique aperture with a more pointed basal tooth. Furthermore, there is a second broken belt between the main belt and the umbilicus in *L.pewzowi*, which is not present in any specimens of this species. *Laeocathaicapotanini* has a more scalariform, uniformly light brown shell, and the basal tooth (when present) is situated closer to the columella than in *L.carinalis*. *Laeocathaicaamdoana* is also similar in colouration, but it is larger, has a blunter keel, a weaker sculpture, and its whorls are never scalariform.

**Figure 11.**
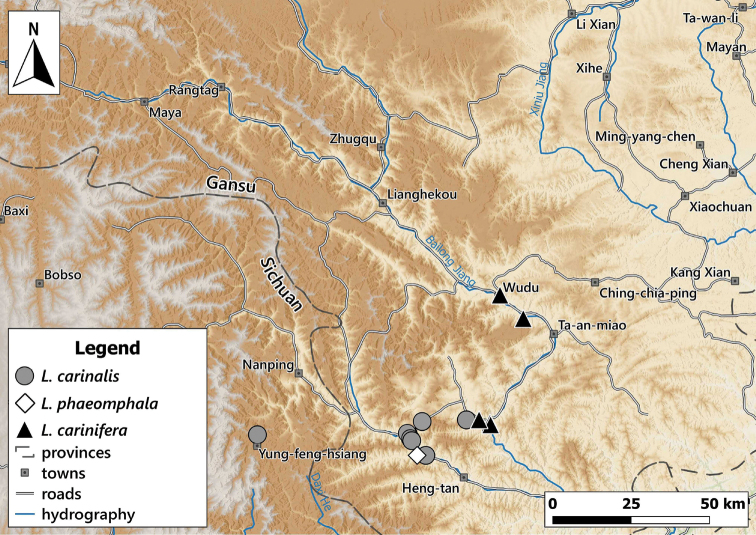
Distribution of *Laeocathaica* species in China (detail from Fig. 2A).

##### Distribution.

Most precise locality data are from the rocky area along the Baishui River, whereas one sample was collected on the bank of the Yangtang River (Fig. 11). The type locality is situated ca. 50 km west in a straight line.

##### Remarks.

We here provide a redescription, an updated differential diagnosis, and notes on the differences between different populations (Table 2).

#### ﻿ Laeocathaica carinifera

Taxon classificationAnimaliaStylommatophoraBradybaenidae

(H. Adams, 1870)

70BD0497-A2E5-5F88-AADC-C96241694A93

Figure 12

Helix (Plectotropis) christinae var. carinifera H. Adams, 1870: 377.
Helix subsimilis Deshayes, 1874: 10, pl. 2, figs 28–29.
Helix christinae . – Möllendorff 1884: 351.
Helix subsimilis . – Gredler 1884: 264.
Helix christinae var. carinifera . – Möllendorff 1884: 351.
Laeocathaica subsimilis . – Möllendorff 1899: 89.
Laeocathaica stenochone Möllendorff 1899: 91, pl. 5, fig. 4. **new synonym**
Laeocathaica subsimilis subsimilis . – Yen 1939: 148, pl. 15, fig. 28.
Laeocathaica stenochone . – Yen 1939: 148, pl. 15, fig. 30.
Laeocathaica (Laeocathaica) stenochone . – Zilch 1968: 175.
Laeocathaica (Laeocathaica) subsimilis . – Zilch 1968: 175.
Laeocathaica subsimilis . – Chen and Zhang 2004: 313, fig. 299 (treats *filippina* as a synonym).
Laeocathaica stenochone . – Chen and Zhang 2004: 314, fig. 301.
Laeocathaica subsimilis subsimilis . – Wu 2004: 86, 89, 98, 112, fig. 17 (figure labelled as L.filippina).

##### Type material.

**Figure 12.**
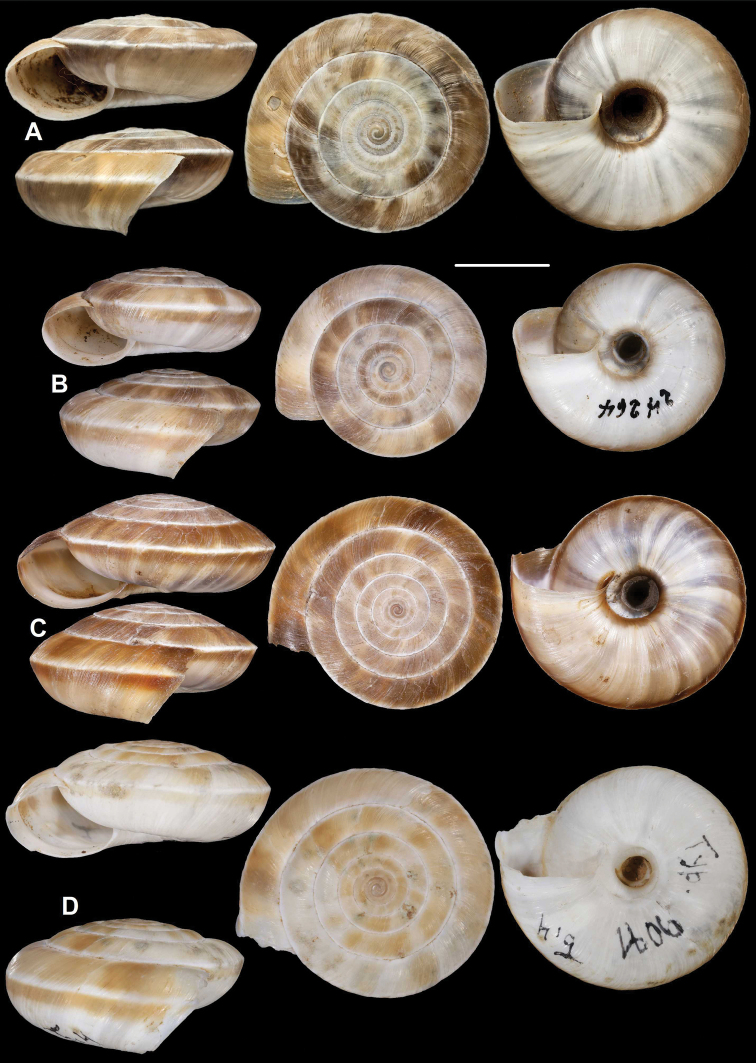
*Laeocathaicacarinifera* (H. Adams, 1870) **A** syntype, NHMUK 1870.7.16.7 **B**SMF 24264 **C** 2016/70a **D** lectotype of *L.stenochone* (SMF 9071). Scale bar: 10 mm. Photographs: B. Páll-Gergely (**C, D**), Kevin Webb, NHM (**A**).

China, Woushan, coll. Swinhoe, NHMUK 1870.7.16.7 (3 shells, probably syntypes of Helixchristinaevar.carinifera, labelled as “christinae var”) (Fig. 12A) • China, coll. Swinhoe, NHMUK 1870.7.16.8 (3 shells, probably syntypes of Helixchristinaevar.carinifera, labelled as “christinae var”) • Thibet, leg. Abbé David, 1870, MNHN/1 syntype of *H.subsimilis* (broken) • Thibet (Moupin), leg. Abbé David, 1869, MNHN/12 syntypes of *H.subsimilis* (some of them are juvenile/broken) • Chine, leg. Abbé David, 1874, MNHN/1 syntype of *H.subsimilis* • China (SO-Gansu): Moupin, Thibet Oriental, leg. David, coll. Deshayes, 1872 in coll. Crosse, MNHN-IM-2014-7944/2 syntypes of *H.subsimilis* • Hsi-gu-tseng, coll. Möllendorff ex coll. Potanin 577, SMF 9071 (lectotype of *L.stenochone*, Fig. 12D) • Same data, SMF 9072/1 (paralectotype of *L.stenochone*) • SO-Gansu, Zw. Yü-lin-guan u. Wen-hsien, SMF 8951/1 (paralectotype of *L.stenochone*) • Sy-tchuan, coll. Möllendorff ex coll. Berezowski, 908c, SMF 24270/1 (paralectotype of *L.stenochone*).

**Table 2.** Shell morphological traits of *Laeocathaicacarinalis* Chen & Zhang, 2004 populations.

Locality no.	Shell diameter	Belt below keel	White belt on keel	Dorsal side	Denticle
2016/64	20.2	medium	thin	flat/not scalariform	strong
2016/70b	20.3–21.5	thin	thick	domed/scalariform	only low thickening
2016/79	19.1–20.3	thick	moderate	flat/scalariform	strong
2016/81	19.8–20.4	thick	moderate	flat/scalariform	1 out of 5 shells
2016/82	18.6–19.8	medium	thin	flat/scalariform	present
2016/83	21.2–22.9	thick	moderate	moderately domed/slightly scalariform	only low thickening

##### Museum material.

China, Yangtze-Tal, coll. Jetschin ex coll. Beddome, SMF 95020/1 (mixed sample with *L.christinae*) • Lü-feng-kou b. Guan-yüan, coll. Möllendorff ex coll. Potanin 270, SMF 24264/4 (Fig. 12B) • China (Sy-tchuan): zw. Guan-yüan u. Dshau-hoa, coll. Möllendorff ex coll. Potanin 275, SMF 24256/3 • WM-China, Sy-tchuan, Chung-King, coll. O. Boettger ex coll. Möllendorff, SMF 24262/4 • China: Badung, Hubei, coll. Möllendorff ex coll. L. Fuchs, SMF 24259/2; O-Sy-tschuan, coll. Möllendorff, SMF 24255/4 • China: W-Hupé, ex Gredler, SMF 294293/2 • China: Kwan-juön-hszién (Kuan-yuan-hsien), Prov. Sze-csuen, China, ex coll. T. Kormos, SMF 24260/1 • China, Coll. H. Rolle, SMF 294294/1; China: Sytchuan, coll. C. Bosch ex coll. H. Rolle, SMF 294292/2 (mixed sample with *L.filippina*) • W-China: Prov. Sy-chuan, coll. C.R. Boettger, SMF 95117/1 • Sytschuan: Tal des Lu-fyn-kou nördlich von der Stadt Juanj-juanj, leg. Potanin, coll. Jetschin, SMF 95116/1 • Same locality, MNHN-IM-2014-7935/2 • China: Yang-dsy Gebiet, coll. Möllendorff ex coll. Heude, SMF 24265/2 • China: Shen-hsi, Liu-ba-ting, coll. Möllendorff ex coll. Potanin 451, SMF 24261/2 • O-Sytchuan, coll. Kobelt (alte Schau-Sammlung) ex coll. Möllendorff, SMF 24258/3 • O-Sy-tchuan, coll. Möllendorff ex coll. B. Schmacker, SMF 24257/4 • Paoning, Szechuan, Don: Tomlin, 1946, MNHN-IM-2014-7931/4 adult + 2 juvenile shells • Chine, Chungking, Sytschouan, coll. Letellier, 1949, MNHN-IM-2014-7936/1 • Chine, coll. Denis, 1945, MNHN-IM-2014-7937/2 • China, Baoning, MNHN-IM-2014-7941/1 juvenile shell • Turkestan, leg. Potanin, MNHN-IM-2014-7943/1 adult + 1 juvenile shell • Chine, collector’s name not readable, 1878, MNHN-IM-2014-7938/1 • Chine, coll. Fischer, MNHN/1 • Hupé, China, MNHN-IM-2014-7940/1 • Hupé, China, coll. Staadt, 1969, MNHN-IM-2014-7929/1 + 2 *L.filippina* shell (mixed sample, erroneous locality for *L.carinifera*).

##### New material.

China • 3 shells; Gansu, Longnan Shi, Wenxian, Bikou Zhen, above the hydroelectric power plant, northern side of Bailong He (locality code: 2016/63); 32°45.966'N, 105°13.005'E; 28 May 2016; A. Hunyadi leg.; HA • 1 shell; Gansu, Longnan Shi, Wenxian, Jianshan Xiang, 1800 m west of Jianshan towards Diaohuya, right side of road no. 212 (locality code: 2016/70b); 33°2.922'N, 104°50.840'E; 29 May 2016; A. Hunyadi leg.; HA • 3 shells; Gansu, Longnan Shi, Wenxian, Jianshan Xiang, southern edge of Hekou Cun, western bank of Bailong He (locality code: 2016/67); 33°02.014'N, 104°53.478'E; 800 m a.s.l.; 29 May 2016; A. Hunyadi leg.; HA • 3 shells; Gansu, Longnan Shi, Wenxian, Jianshan Xiang, 600 m west of Jianshan towards Diaohuya (locality code: 2016/70a); 33°02.559'N, 104°51.254'E; 850 m a.s.l.; 29 May 2016; A. Hunyadi leg.; HA (Fig. 12C) • 3 shells; Gansu, Longnan Shi, Wenxian, Jianshan Xiang, 1200 m south of Hekou Cun, eastern bank of Bailong He (locality code: 2016/68); 33°01.703'N, 104°53.602'E; 810 m a.s.l.; 29 May 2016; A. Hunyadi leg.; HA • 2 shells; Gansu, Longnan Shi, Wudu Xian, Hanwang Zhen, Wanxiangdong, path below the cave (locality code: 2016/85); 33°19.824'N, 105°00.273'E; 1160 m a.s.l.; 01 June 2016; A. Hunyadi leg.; HA • 2 shells; Shaanxi, Hanzhong Shi, Lueyang Xian, Gaojiaba, along the highway (locality code: 2016/96); 33°22.090'N, 106°10.116'E; 06 June 2016; A. Hunyadi leg.; HA • 3 shells; Gansu, Longnan Shi, Wudu Xian, Chengguan Zhen, northwest of Jiezhou Botanical Garden, hill above the city (locality code: 2016/86); 33°23.809'N, 104°55.524'E; 1035 m a.s.l.; 01 June 2016; A. Hunyadi leg.; HA • 2 shells; Sichuan, Chengdu Shi, Chengdu, Nanda Jie, stone fence (locality code: 2015/78); 30°39.228'N, 104°3.659'E; 23 June 2015; A. Hunyadi & M. Szekeres leg.; HA.

##### Distribution.

The original description Helixchristinaevar.carinifera was published together with that of the nominotypical form, without any specification of a type locality. However, on one of the boxes of var.carinifera in the NHM, the locality Woushan (probably Wushan, Chongqing at 31°5'N, 109°53'E) was mentioned. The type locality of *Helixsubsimilis* is Moupin (Baoxing, at 30°22'N, 102°49'E) in Tibet. Furthermore, Wu (2004) dissected *Laeocathaicasubsimilis* specimens collected at Nanchong, Sichuan. All precise locality data are known from southern Gansu and from the centre of Chengdu city in Sichuan (probably introduced?). Therefore, it is possible that *L.carinifera* is a widespread species, or the locality data from 250–450 km from southern Gansu are the results of imprecise labelling. On the map (Fig. 11) we only indicate the newly reported samples from southern Gansu.

##### Remarks.

Adams (1870) described *Helixchristinae* and Helixchristinaevar.carinifera . According to the original description, var.carinifera differs from the nominotypical form by the smaller shell, the more acute keel, and the narrower umbilicus. We found a sample labelled *Helixchristinae* and two labelled as “*Helixchristinae* var.” in the NHM. The latter two samples differ from the former one exactly in the traits mentioned by Adams. Thus, although they are not labelled as var.carinifera, it is clear that they represent syntypes of that taxon.

The syntypes of Helixchristinaevar.carinifera are identical with the types of *Helixsubsimilis*, and thus, the latter is a junior synonym of the former. Although both Möllendorff (1884) and Gredler (1884) synonymised *H.subsimilis* with *H.carinifera*, this species (*L.carinifera*) has been mentioned in the literature as *Laeocathaicasubsimilis*. *Laeocathaicastenochone* is also identical with both Helixchristinaevar.carinifera and *Helixsubsimilis*, and therefore, it is also treated as a junior synonym.

#### ﻿ Laeocathaica christinae

Taxon classificationAnimaliaStylommatophoraBradybaenidae

(H. Adams, 1870)

B8FBBFF7-CEB4-5DCF-A96A-C68A5F6A68F0

Figure 13

Helix (Plectotropis) christinae H. Adams, 1870: 377, pl. 27, figs 4, 4a.
Helix christinae . – Gredler 1884: 264.
Laeocathaica christinae . – Möllendorff 1899: 88.
Laeocathaica (Laeocathaica) christinae christinae . – Zilch,1968: 173.
Laeocathaica christinae . – Chen and Zhang 2004: 334, fig. 326.

##### Type material.

**Figure 13.**
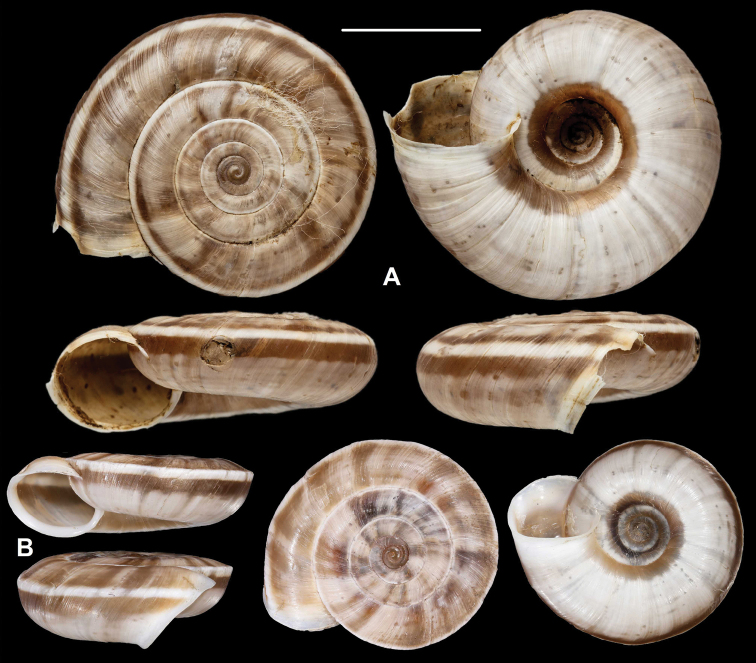
Shell of *Laeocathaicachristinae* (H. Adams, 1870) **A** probable syntype, NHMUK 1870.7.16.6 **B** 2010/29. Scale bar: 10 mm. Photographs: B Páll-Gergely (**B**), Kevin Webb, NHM (**A**).

China, coll. Swinhoe, NHMUK 1870.7.16.6 (3 shells, probably syntypes, Fig. 13A) • Chine, Ichang & Fungsiang, Achat Sallé, MNHN-IM-2014-7932/3 (probably syntypes).

##### Museum material.

Asia Centrale, MNHN-IM-2014-7933/3 • Asia Centrale, MNHN-IM-2014-7934/7 • Hupé, China, coll. Staadt, 1969, MNHN-IM-2014-7929/2 + 1 *L.carinifera* shell (mixed sample, erroneous locality for *L.carinifera*) • Moupin, leg. Abbé David, coll. Deshayes, 1872, MNHN-IM-2014-7945/2 (probably erroneous locality).

##### New material.

China • 10 shells; Hubei, Enshi Tujiazu Miaozu Zizhizhou, Badong Xian, east of Badong, Bashan Senlin Gongyuan, (next to Xinlingzhen) (locality code: 2010/29); 31°01.472'N, 110°25.284'E; 225 m a.s.l.; 03 November 2010; A. Hunyadi leg.; HA (Fig. 13B).

##### Distribution.

**Figure 14.**
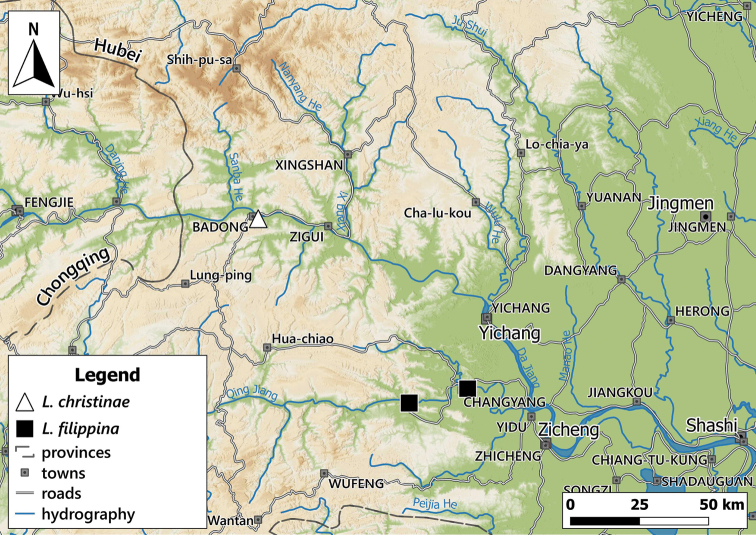
Distribution of *Laeocathaica* species in China (**B** in Fig. 2).

The type localities and the newly collected sample suggest that this species lives more upstream in the Yangtze valley than *L.filippina*. The samples from Moupin (Baoxing County, Sichuan) are probably erroneous. We indicated only the newly collected sample on the map (Fig. 14).

##### Remarks.

We found three samples in the NHM. One of them, containing two shells, was labelled *Helixchristinae*. The other two lots, labelled “*Helixchristinae* var.”, contained three shells each. The latter two samples are probably syntypes of Helixchristinaevar.carinifera, described in the same publication (Adams 1870; see further details under that species). None of the two *Helixchristinae* shells (NHMUK 1870.7.16.6) are identical with the shells figured in the original description (Adams 1870: pl. 27, figs 4, 4a). However, the indication of the collector (Swinhoe) agrees with the collector mentioned in the original description. Thus, we treat the two shells of that lot as syntypes.

Adams (1870) gave two type localities: “Ichang and Fungsiang gorges, China”. The former is situated upstream of Yichang City in Hubei (30°56'N, 110°48.7'E), whereas the latter is Fengxiangxia, Fengjie County in Chongqing (31°2'N, 109°35'E). Our sample 2010/29 is geographically located between the two type localities. The shells of this sample are identical with the types, but they are smaller.

#### ﻿ Laeocathaica dejeana

Taxon classificationAnimaliaStylommatophoraBradybaenidae

(Heude, 1882)

D06BDA39-0FF4-5DD4-B4E3-8240F85C80F3

Figure 15

Helix dejeana Heude, 1882: 21, pl. 20, fig. 17.
Cathaica (Campylocathaica) dejeana . – Chen and Zhang 2004: 270–272, fig. 255.
Laeocathaica dejeana . – Chen and Zhang 2004: 337, fig. 330.

##### Type material.

According to Johnson (1973) there is a paratype in the USNM (inv. number: 472128), which was not examined by us.

**Figure 15.**
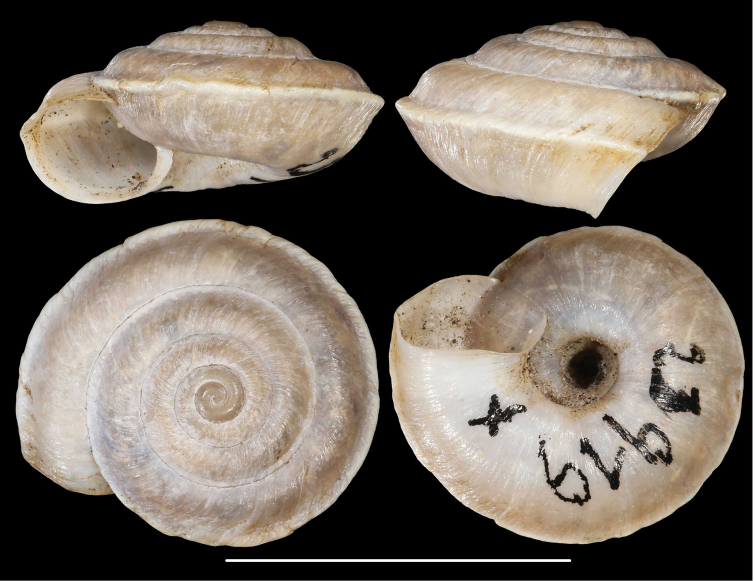
*Laeocathaicadejeana* (Heude, 1882), SMF 23919. Scale bar: 10 mm. All photographs: B. Páll-Gergely.

##### Museum material.

China: Da-tshien-lu am Ya-lung, coll. Möllendorff ex coll. Potanin 380, SMF 23919/4 (Fig. 15) • Sy-tschuan, Umgebungen der Stadt Da-zsjan-lu, det. Möllendorff, MNHN-IM-2014-7946/1.

#### ﻿ Laeocathaica dityla

Taxon classificationAnimaliaStylommatophoraBradybaenidae

Möllendorff, 1899

57EEB676-18ED-5EE0-AF81-D0569A38DFFA

Figure 16

Laeocathaica dityla Möllendorff, 1899: 99–100, pl. 6, fig. 8.
Laeocathaica dityla . – Yen 1939: 149, pl. 15, fig. 42.
Laeocathaica (Laeocathaica) dityla . – Zilch 1968: 174.
Laeocathaica dityla . – Chen and Zhang 2004: 332, fig. 324.

##### Type material.

**Figure 16.**
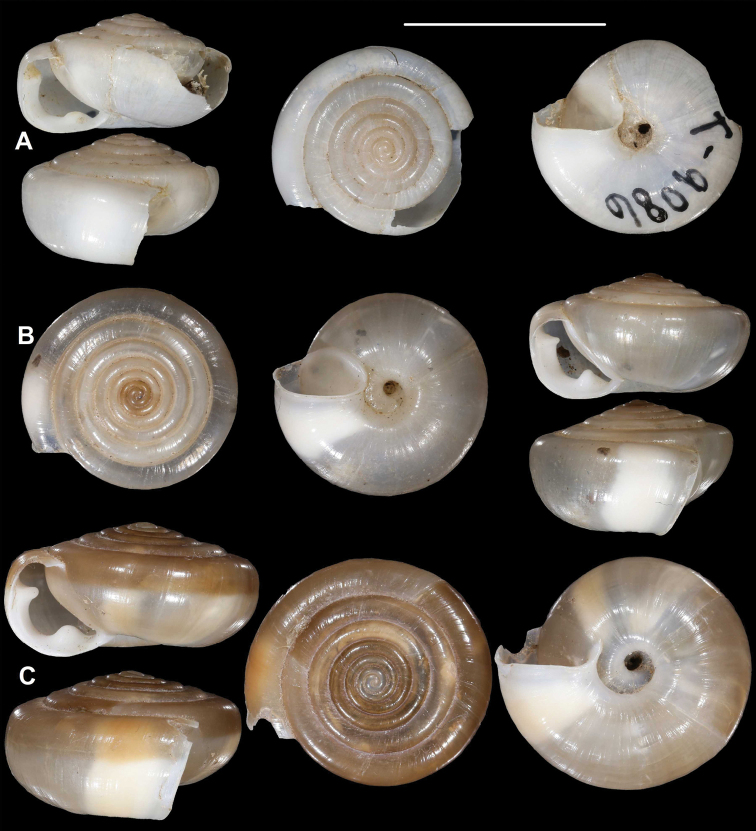
*Laeocathaicadityla***A** lectotype (SMF 9086) **B** 2016/84 **C** 2016/88. Scale bar: 10 mm. All photographs: B. Páll-Gergely.

SO-Gansu, zw. Li-tshia-pu u. Hsi-gu-tsheng, coll. Möllendorff ex coll. Potanin 776, SMF 9086 (lectotype, Fig. 16A) • Same data, SMF 9087 (paralectotype); Tshiu-dsei-dsy, coll. Möllendorff ex coll. Potanin 22, SMF 9088/1 (paralectotype).

##### New material.

China • 4 shells; Gansu, Longnan Shi, Dangchang Xian, Guanting Zhen, 1.5 km north of Guanting towards Dangchang (locality code: 2016/88); 33°50.803'N, 104°32.470'E; 1815 m a.s.l.; 02 June 2016; A. Hunyadi leg.; HA (Fig. 16C) • 5 shells; Gansu, Longnan Shi, Wudu Xian, Hanwang Zhen, Wanxiangdong, serpentine leading to the cave (locality code: 2016/84); 33°20.383'N, 104°59.876'E; 1010 m a.s.l.; 01 June 2016; A. Hunyadi leg.; HA (Fig. 16B) • 1 shell; Gansu, Longnan Shi, Dangchang Xian, Lianghekou Xiang, eastern edge of Lianghekou Cun (locality code: 2016/90); 33°41.587'N, 104°29.379'E; 02 June 2016; A. Hunyadi leg.; HA • 9 shells; Gansu, Longnan Shi, Wudu Xian, Jiaogong Zhen, 1.5 km west of Chenjiaba Cun, Zhaoyangdong, below the cave (locality code: 2016/95); 33°31.924'N, 104°39.286'E; 1175 m a.s.l.; 04 June 2016; A. Hunyadi leg.; HA • 1 shells; Gansu, Longnan Shi, Wudu Xian, Chengguan Zhen, northwest of Jiezhou Botanical Garden, hill above the city (locality code: 2016/86); 33°23.809'N, 104°55.524'E; 1035 m a.s.l.; 01 June 2016; A. Hunyadi leg.; HA • 2 shells; Gansu, Dangchangxian, Shawanxiang, 401 km point along R212 (locality code: 20110422C); 33°38.067'N, 104°33.240'E; 1258 m a.s.l.; 21 April 2011; Y. Nakahara, K. Okubo & K. Otani leg.; PGB.

##### Distribution.

**Figure 17.**
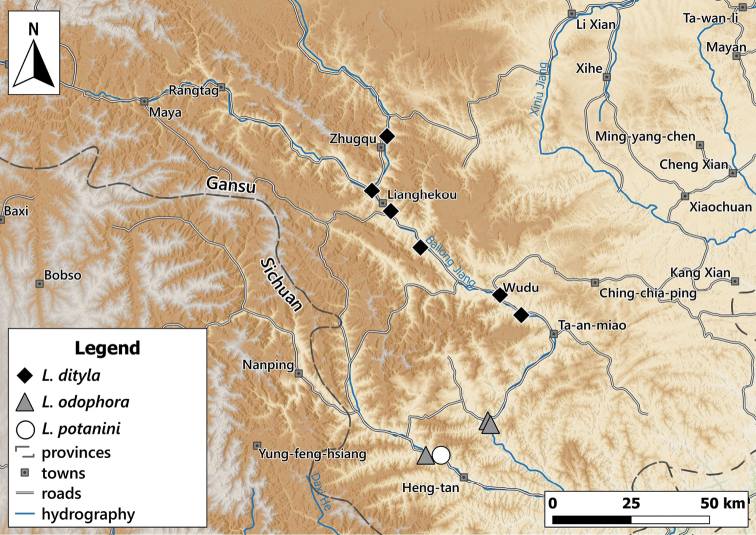
Distribution of *Laeocathaica* species in China (**A** in Fig. 2).

New samples were collected along the Bailong and Minjiang rivers (Fig. 17).

#### ﻿ Laeocathaica dolani

Taxon classificationAnimaliaStylommatophoraBradybaenidae

(Pilsbry, 1934)

D34CEA28-5153-5B4D-AD61-571F0FD7784E

Figure 18A

Cathaica (Laeocathaica) dolani Pilsbry, 1934: 16, pl. 3, figs 4, 4a–c.
Laeocathaica dolani . – Chen and Zhang 2004: 335, fig. 328.

##### Type material.

**Figure 18.**
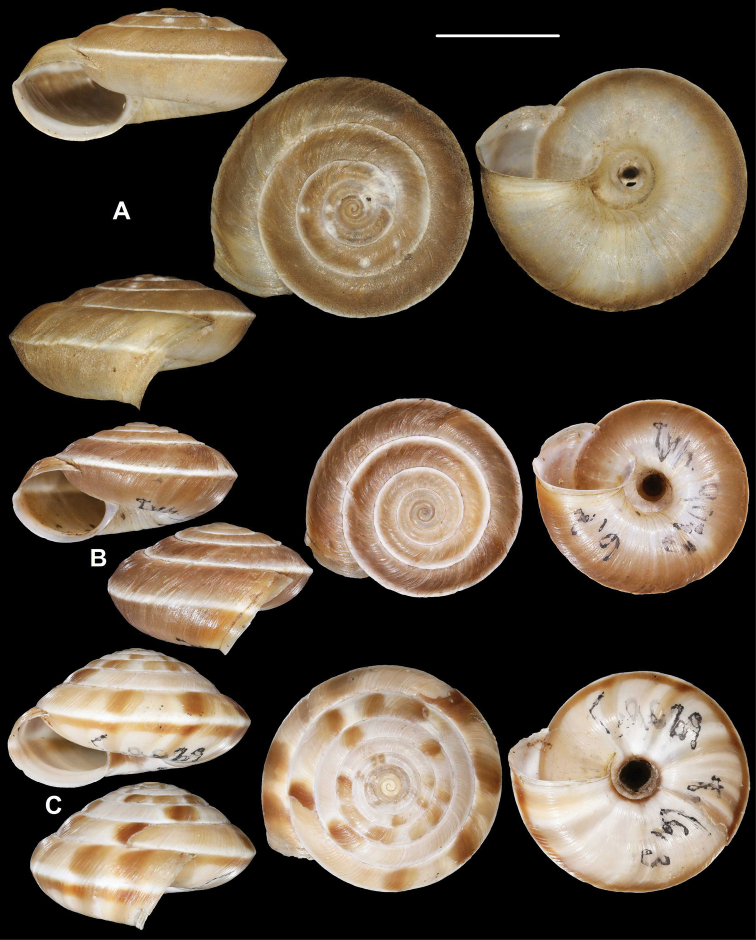
Shells of *Laeocathaica* species **A***Laeocathaicadolani* Pilsbry, 1934 (holotype, ANSP 162061) **B***Laeocathaicaleucorhaphe* (Möllendorff, 1899), lectotype (SMF 9073) **C***Laeocathaicaphaeomphala* Möllendorff, 1899, lectotype (SMF 9089). Scale bar: 10 mm. All photographs: B. Páll-Gergely.

China, Szechuan, Romichengu, Brooke Dolan, W. China Expedition 1931, ANSP 162061 (holotype in Fig. 18A + 3 paratypes).

#### ﻿ Laeocathaica filippina

Taxon classificationAnimaliaStylommatophoraBradybaenidae

(Heude, 1882)

E5C9E285-D0C4-5ABB-9A62-F7BEED3178A4

Figure 19

Helix filippina Heude, 1882: 23, pl. 20, fig. 19.
Helix (Plectopylis) subchristinae Ancey, 1882: 44.
Helix subsimilis var. filippina . – Gredler 1884: 264.
Laeocathaica filippina . – Möllendorff 1899: 88–89.
Laeocathaica subsimilis filippina . – Yen 1939: 148, pl. 15, fig. 29.
Laeocathaica (Laeocathaica) christinae filippina . – Zilch 1968: 173.

##### Type material.

**Figure 19.**
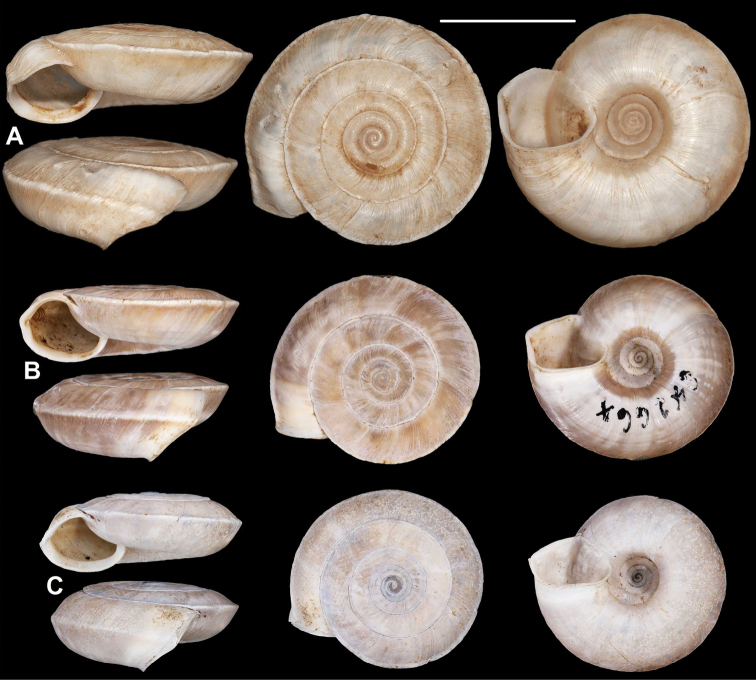
Shells of *Laeocathaicafilippina* (Heude, 1882) **A** syntype, USNM 427127 **B**SMF 24266 **C** 2010/25. Scale bar: 10 mm. Photographs: downloaded from the webpage of USNM (**A**), B. Páll-Gergely (**B–C**).

Patong, Heude type coll., USNM 472127, (1 syntype, Fig. 19A).

##### Museum material.

China, W-Hupei, coll. K. Hashagen, SMF 24226/3 • Hubei, Badung, coll. Möllendorff, SMF 24266/4 (Fig. 19B) • China: Badung, Hubei: coll. Kobelt (alte Schau-Sammlung) ex coll. Möllendorff, SMF 24266a/3 • W-Hupei, China, coll. Naegele ex coll. Gredler 1906, SMF 50089/2 (labelled as *L.filippina*, det. Wu 2008) • China, SMF 24227/1 (labelled as *L.subsimilis*, det. Wu 2008) • China, Sytchuan, Changyang, coll. O. Boettger ex coll. B. Schmacker 1893, SMF 24267/2 • China: Badung, Hubei: coll. C.R. Boettger 1904, SMF 95118/5 • China: Changyang, coll. C. Bosch ex coll. Sowerby ex coll. Fulton, SMF 294296/3 • China: Changyang, coll. Ehrmann ex coll. Sowerby ex coll. Fulton, SMF 294295/1 • Changyang, Coll. Denis 1945, MNHN-IM-2014-7942/2 • China: Sytchuan, coll. C. Bosch ex coll. H. Rolle, SMF 294292/1 (mixed sample with *L.carinifera*).

##### New material.

China • 7 shells; Hubei, Yichang Shi, Changyang Tujiazu Zizhixian, Qingjiang Hualang Fengjingqu, Geheyan Shuiku, Wuluozhougli Shan (locality code: 2010/25); 30°25.805'N, 110°59.254'E; 260 m a.s.l.; 31 October 2010; A. Hunyadi leg.; HA (Fig. 19C) • 3 broken shells; Hubei, Yichang Shi, Changyang Tujiazu Zizhixian, eastern edge of Changyang, environment of Hukouwan, rocks around the budhist temple (locality code: 2010/23); 30°28.622'N, 111°12.421'E; 95 m a.s.l.; 31 October 2010; A. Hunyadi leg.; HA.

##### Distribution.

The newly collected samples, and the several museum samples from Changyang (at 30°28'N, 111°12'E), suggest that this species lives downstream along the river Yangtze compared to *L.christinae* (Fig. 13). The type locality of *L.filippina* (Badong) is situated more upstream, within the area of *L.christinae*. However, it may be erroneous, as at the time of the description the nearest large city was usually mentioned on the labels.

##### Remarks.

We did not examine the types of *Helixsubchristinae* Ancey, 1882, and treat it as a synonym of *L.christinae* following Gredler (1884), while Richardson (1983) listed it under *L.christinae*.

#### ﻿ Laeocathaica hisanoi

Taxon classificationAnimaliaStylommatophoraBradybaenidae

Páll-Gergely sp. nov.

7F75939E-72C5-547A-9F9E-2F3491F4A6DE

http://zoobank.org/F273CAC1-E575-426C-ACA6-A16F6CB80171

Figure 20D

##### Type material.

**Figure 20.**
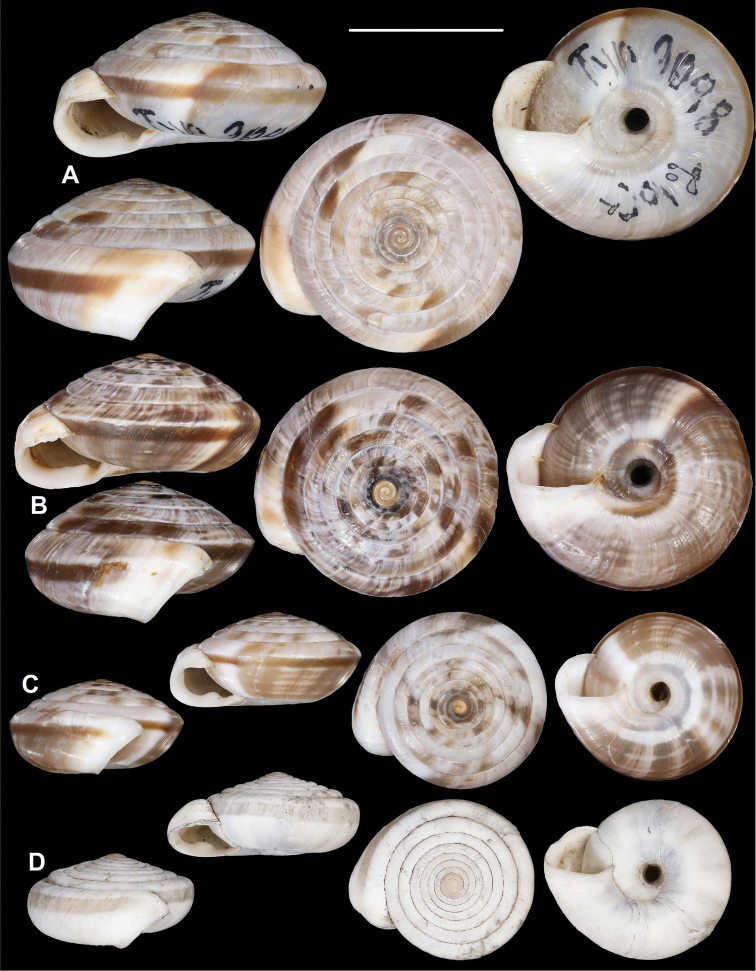
*Laeocathaica* species **A***Laeocathaicapolytyla* Möllendorff, 1899, lectotype (SMF 9198) **B***Laeocathaicapolytyla*, 2016/78 **C***Laeocathaicapolytyla*, 2016/65 **D***Laeocathaicahisanoi* sp. nov. (holotype, SMF 336708). Scale bar: 10 mm. All photographs: B. Páll-Gergely.

***Holotype*** China • S Kansu, China, coll. S. Hisano, 24.05.204, SMF 336708 (D: 11.6 mm, H: 5.2 mm) (Fig. 20D).***Paratype*** China • Same data as for holotype; SMF 363469.

##### Diagnosis.

A small *Laeocathaica* species with many (8.5) whorls, conical dorsal side, rounded body whorl and single, small basal tooth that is situated close to the columella.

##### Description.

Shell sinistral, depressed, dorsal side conical with protruding apex, body whorl shouldered; colour chalk white with a single brownish belt below shoulder; entire shell consists of 8.5 whorls, protoconch consists of 1.75–2 whorls, very finely granulose, conspicuously elevated compared to first teleoconch whorls; teleoconch with fine, irregular growth lines, without any notable sculpture, although both examined shells were corroded; last quarter whorl with slight subsutural furrow; aperture semilunar, very strongly oblique to shell axis; peristome sharp, very slightly expanded dorsally, with thickening situated behind peristome edge; basal tooth blunt, elongated, situated ca. at the middle of basal peristome; parietal callus inconspicuous, appears only as thick calcareous layer; umbilicus open, narrow, shows all whorl, with the last half of body whorl extremely widened, resulting in a “9”-shape.

**Measurements** (in mm): D: 11.5–11.6; H: 5.2–5.3 (*n* = 2).

##### Differential diagnosis.

The most similar species is *L.polytyla*, which is usually larger, has one whorl more, has a more elevated spire with a domed dorsal side, a rounded body whorl, and a comparatively smaller basal tooth situated closer to the columella.

##### Etymology.

This new species is named after S. Hisano, who collected the type material.

##### Distribution.

This new species is known from a single museum sample only, consisting of two shells.

#### ﻿ Laeocathaica leucorhaphe

Taxon classificationAnimaliaStylommatophoraBradybaenidae

Möllendorff, 1899

61C975B3-AE43-5330-9D75-A8D09A1E87E2

Figure 18B

Laeocathaica leucorhaphe Möllendorff, 1899: 95–96, pl. 6, fig. 2.
Laeocathaica leucorhaphe . – Yen 1939: 149, pl. 15, fig. 36.
Laeocathaica (Laeocathaica) leucorhaphe . – Zilch 1968: 174.
Laeocathaica leucorhaphe . – Chen and Zhang 2004: 323, fig. 312.

##### Types examined.

N-Sytchuan: am Tung-ho, coll. Möllendorff ex coll. Potanin 312b, SMF 9073 (lectotype, Fig. 18B).

#### ﻿ Laeocathaica minwui

Taxon classificationAnimaliaStylommatophoraBradybaenidae

Páll-Gergely sp. nov.

083421BC-C53C-5D7A-9A2A-77EA57F4A71F

http://zoobank.org/C96CE473-3692-4306-9AB2-2AEFDA307824

Figure 21

##### Type material.

**Figure 21.**
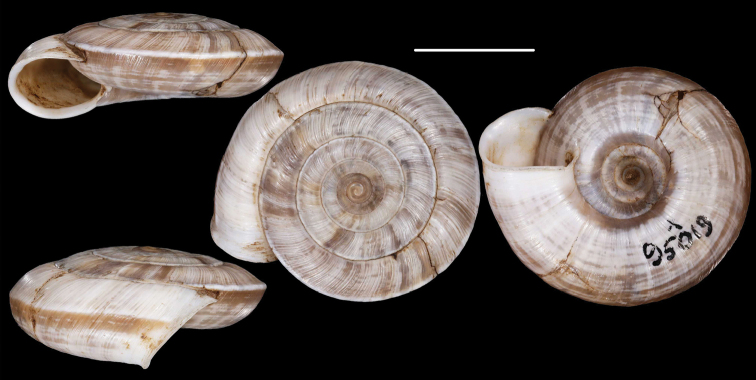
*Laeocathaicaminwui* sp. nov. (holotype, SMF 95019). Scale bar: 10 mm. Photographs: B. Páll-Gergely.

***Holotype*** China • China, O. Sy-tshuan, coll. C.R. Boettger ex coll. Möllendorff ex coll. L. Fuchs, SMF 95019 (D = 23.1, H = 9.1) (Fig. 21). ***Paratypes*** China • Yangtze-Tal, coll. Jetschin ex coll. Beddome, SMF 95020/1 (mixed sample with *L.carinifera*) • W. China, Sy-tshuan, coll. Kobelt ex coll. Möllendorff, SMF 6920/1 (det. Wu 2008, labelled as *L.christinae*) • China: Hupei: Kao-cha-hien, coll. O. Boettger ex coll. B. Schmacker, 1893, SMF 24263/1 (det. Wu 2008, labelled as *L.christinae*) • China, O. Sy-tshuan, coll. Möllendorff, SMF 24255a/5 • China: Chang-Yang, coll. O. Boettger ex coll. M. Schmacker, SMF 42563/2 • Moupin, leg. Abbé David, MNHN-IM-2014-7939/14 (probably erroneous locality).

##### Diagnosis.

A rather large *Laeocathaica* species with a sharp keel, a domed dorsal side, an oval aperture and a marmorated ventral side.

##### Description.

Shell sinistral, depressed, with domed dorsal side, keel strong, situated in the middle of body whorl, whitish; dorsal side latte-coloured, with darker and paler areas alternating as the shell grows; ventral side with white and pale brownish (latte) spiral bands forming a marmorated colour pattern; inner side of umbilicus with brownish spiral band; protoconch light brownish, ca. 1.5 whorls, finely granulose, slightly protrudes above first whorls of teleoconch; entire shell consists of six whorls; dorsal side finely ribbed, ventral side smoother, only with growth lines; umbilicus ca. one third of shell width; shows all whorls; periumbilical keel absent; aperture oblique to shell axis, oval, without incision at the position of keel; peristome white, expanded and slightly thickened, but not reflexed (only in direction of umbilicus); parietal callus practically absent, only with some additional translucent calcareous layer.

**Measurements (in mm)**: D = 23.1, H = 9.1 (holotype).

##### Differential diagnosis.

*Laeocathaicaminwui* sp. nov. has been confused with *L.christinae* in museum collections, probably due to the lack of examination of the types of *L.christinae*. However, *L.christinae* has a flatter dorsal side, a more upper-situated peripheral keel, a darker brown (instead of latte) colour, a more uniformly white ventral side with a brown spiral band inside the umbilicus, and brownish spots. In contrast, in the new species the ventral side is characterised by a marmorated (marbled-like) pattern resulted by the fusing of whitish and pale brown spiral bands. *Laeocathaicafilippina* has a notched aperture at the position of the peripheral keel, a more brownish colour, and a less marmorated ventral side. See also Table 3.

**Table 3.** Differences between *L.carinifera*, *L.christinae*, *L.filippina*, and *L.minwui* sp. nov.

Species	Dorsal surface	Keel on body whorl	Aperture (palatal part)	Colour of dorsal side	Ventral side
*L.carinifera*	domed	acute, in the middle of body whorl	rounded	lighter and darker brownish patches alternate rather abruptly resulting in a mosaic-like structure	umbilicus narrow, colour pale, or light and darker stripes alternate
*L.christinae*	nearly flat/ slightly domed	blunt to acute, upper part of body whorl	rounded	same as in *carinifera*, just brown colour darker and there is usually a brown and a white spiral band	umbilicus wider, mostly white with slender darker radial stripes and dark dots
*L.filippina*	domed	acute, upper part of body whorl	notched	paler than *carinifera* and *christinae*, light and brown patches alternate smoothly	umbilicus wider, similar to *minwui* sp. nov., but paler and less nicely marmorated
*L.minwui* sp. nov.	domed	acute, upper part of body whorl	rounded	mostly cream/latte-coloured, lighter and darker patches alternate smoothly	umbilicus wider, latte and white spiral bands form a marmorated pattern

##### Etymology.

This new species is dedicated to and named after Dr. Min Wu, the leading expert of Chinese Camaenidae.

##### Distribution.

This new species is only known from historical samples from the Yangtze valley. Other samples labelled as being collected from Sichuan are not precise enough to understand their geographic origin.

#### ﻿ Laeocathaica odophora

Taxon classificationAnimaliaStylommatophoraBradybaenidae

Möllendorff, 1899

FCECE8A6-1EAC-5052-A2CC-B74C504847F0

Figure 22

Laeocathaica odophora Möllendorff, 1899: 97–98, pl. 6, fig. 6.
Laeocathaica odophora . – Yen 1939: 149, pl. 15, fig. 39.
Laeocathaica (Laeocathaica) odophora . – Zilch 1968: 174.
Laeocathaica odophora . – Chen and Zhang 2004: 328, fig. 318.

##### Type material.

**Figure 22.**
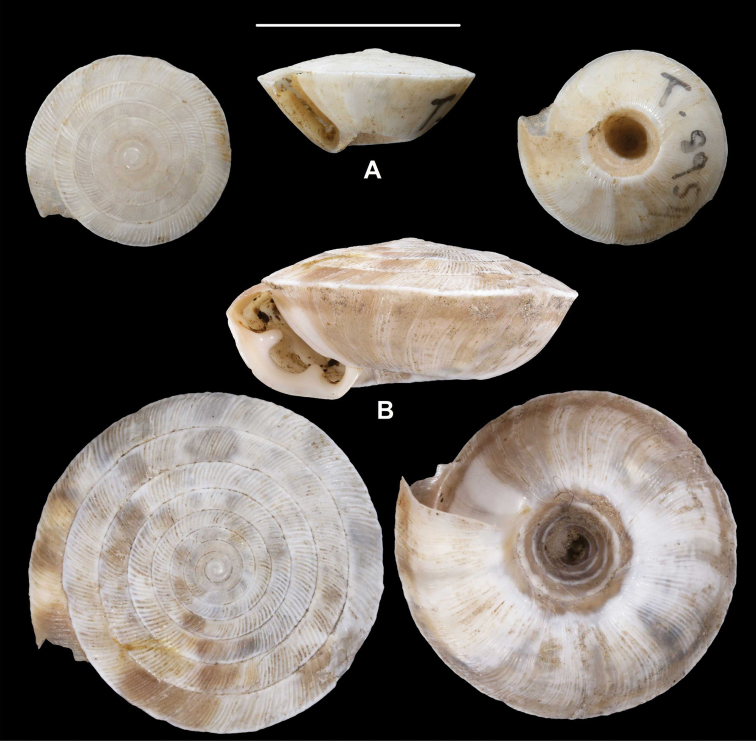
*Laeocathaicaodophora* Möllendorff, 1899 **A** holotype (SMF 8954) **B** 2016/70b. Scale bar: 10 mm. All photographs: B. Páll-Gergely.

S-Gansu, Dshie-dshou, coll. Möllendorff ex coll. Potanin 254, SMF 8954 (holotype = juvenile shell, Fig. 22A).

##### New material.

China • 1 photographed shell; Gansu, Longnan Shi, Wenxian, Jianshan Xiang, 1800 m west of Jianshan towards Diaohuya, right side of road no. 212 (locality code: 2016/70b); 33°2.922'N, 104°50.840'E; 29 May 2016; A. Hunyadi leg.; HA (Fig. 22B) • 5 adult + 2 juvenile shells; Gansu, Longnan Shi, Wenxian, Jianshan Xiang, 1200 m south of Hekou Cun, eastern bank of Bailong He (locality code: 2016/68); 33°01.703'N, 104°53.602'E; 810 m a.s.l.; 29 May 2016; A. Hunyadi leg.; HA • 1 shell; Gansu, Longnan Shi, Wenxian, Jianshan Xiang, western edge of Hekou Cun towards Caojiaba, right side of road no. 212 (locality code: 2016/69); 33°2.343'N, 104°53.045'E; 29 May 2016; A. Hunyadi leg.; HA • 1 juvenile shell; Gansu, Longnan Shi, Wenxian, Chengguan Zhen, next to a museum (locality code: 2016/64); 32°56.471'N, 104°40.379'E; 960–970 m a.s.l.; 28 May 2016; A. Hunyadi leg.; HA.

##### Description.

Shell sinistral, depressed, strongly keeled, dorsal side domed, ventral side conical; shell colour basically brownish, greyish, latte-coloured, with whitish stripes; as a result dorsal surface mosaic-like, ventral side striped; keel always white, there is always a brownish belt just below the keel, periumbilical keel always white; entire shell consists of 9–9.5 whorls; protoconch consists of 1.5 whorls, brownish, seemingly smooth, extremely finely granulose, rather matte; white keels of every whorl slightly elevated from dorsal surface, but dorsal surface almost continuous, suture practically absent; dorsal side with fine, irregular wrinkles (most wrinkles stand alone, but some of them unite to each other); ventral surface with less prominent wrinkles; umbilicus rather narrow, regular funnel-shaped, shows all whorls; periumbilical keel blunt; aperture semilunar, peristome very slightly expanded, but not reflexed or thickened; palatal swelling whitish, with two prominent denticles, situated in some distance from peristome; parietal wall with some whitish thickening in adult shells. Juveniles reverse conical in shape; several apertural barriers are built during lifetime; palatal swelling of juveniles appears as a continuous ridge, although the two denticles recognisable.

##### Distribution.

Known from a few localities in southern Gansu Province (Fig. 17).

##### Remarks.

The single juvenile shell of sample 2016/64 has a narrower, blunter umbilical keel than the holotype, and it is possible that it belongs to another species. However, the juvenile shell of sample 2016/68 is identical with the holotype.

#### ﻿ Laeocathaica pewzowi

Taxon classificationAnimaliaStylommatophoraBradybaenidae

(Möllendorff, 1899)

57271559-08DD-5D51-9D5F-81449392DA7F

Figure 23C, D

Laeocathaica pewzowi Möllendorff, 1899: 98, pl. 6, figs 4, 4a.
Laeocathaica pewzowi . – Yen 1939: 149, pl. 15, fig. 40.
Laeocathaica (Laeocathaica) pewzowi . – Zilch 1968: 174.
Laeocathaica pewzowi . – Chen and Zhang 2004: 329, fig. 320.
Laeocathaica pewzowi . – Schileyko 2004: 1686, fig. 2174a.

##### Type material.

**Figure 23.**
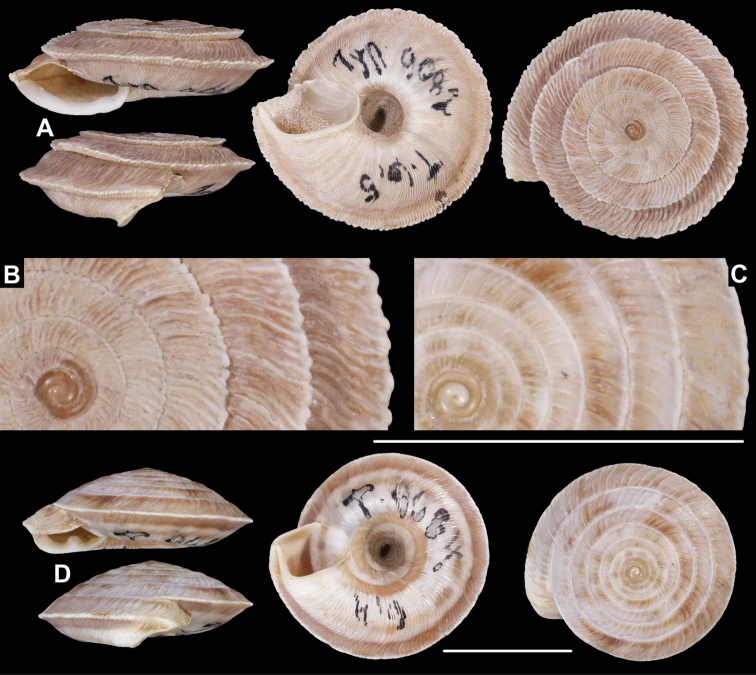
*Laeocathaica* species **A, B***Laeocathaicapotanini* (lectotype, SMF 9082) **C, D***Laeocathaicapewzowi* (lectotype, SMF 9084). Scale bars: 10 mm. All photographs: B. Páll-Gergely.

S-Gansu, Wen-hsien, coll. Möllendorff ex coll. Potanin 248, 661, 793, SMF 9084 (lectotype, Fig. 23C, D) • Same data, SMF 9084/3 (paralectotypes) • Same data, coll. C. Boettger, SMF 9084/1.

##### Museum material.

Nung-dan b. Wen-Hsien, coll. Möllendorff, SMF 24268/1.

#### ﻿ Laeocathaica phaeomphala

Taxon classificationAnimaliaStylommatophoraBradybaenidae

Möllendorff, 1899

54FF3F9D-7C86-5653-AE58-48314408B2E4

Figure 18C

Laeocathaica phaeomphala Möllendorff, 1899: 96, pl. 6, fig. 3.
Laeocathaica phaeomphala . – Yen 1939: 149, pl. 15, fig. 37.
Laeocathaica (Laeocathaica) phaeomphala . – Zilch 1968: 174.
Laeocathaica phaeomphala . – Chen and Zhang 2004: 325, fig. 314.

##### Type material.

S-Gansu, Wenhsien, coll. Möllendorff ex coll. Potanin 51b, 72, 741, SMF 9089 (lectotype, Fig. 18C) • Same data, SMF 9090/3+1 (paralectotypes, one of them from coll. C. Boettger).

##### New material.

China • 6 shells; Gansu, Longnan Shi, Wenxian, Chengguan Zhen, next to a museum (locality code: 2016/64); 32°56.471'N, 104°40.379'E; 960–970 m a.s.l.; 28 May 2016; A. Hunyadi leg.; HA.

#### ﻿ Laeocathaica polytyla

Taxon classificationAnimaliaStylommatophoraBradybaenidae

Möllendorff, 1899

DCB97175-E6ED-5BD8-9083-7D20D840F165

Figure 20A–C

Laeocathaica polytyla Möllendorff, 1899: 98–99, pl. 6, fig. 7.
Laeocathaica polytyla . – Yen 1939: 149, pl. 15, fig. 41.
Laeocathaica (Laeocathaica) polytyla . – Zilch1968: 174.
Laeocathaica polytyla . – Chen & Zhang, 2004: 331, fig. 322.
Laeocathaica polytyla . – Schileyko 2004: 1686, fig. 2174b–d.

##### Type material.

Nan-Ping, Sung-pan, coll. Möllendorff ex coll. Potanin 725b, 744, SMF 9098 (lectotype, Fig. 20A) • Same data, SMF 9099/6 (paralectotypes) • China, S-Gansu, leg. S. Hisano, 22.06.2004, ex coll. S. Hisano, 2011, SMF 336710/1.

**Figure 24.**
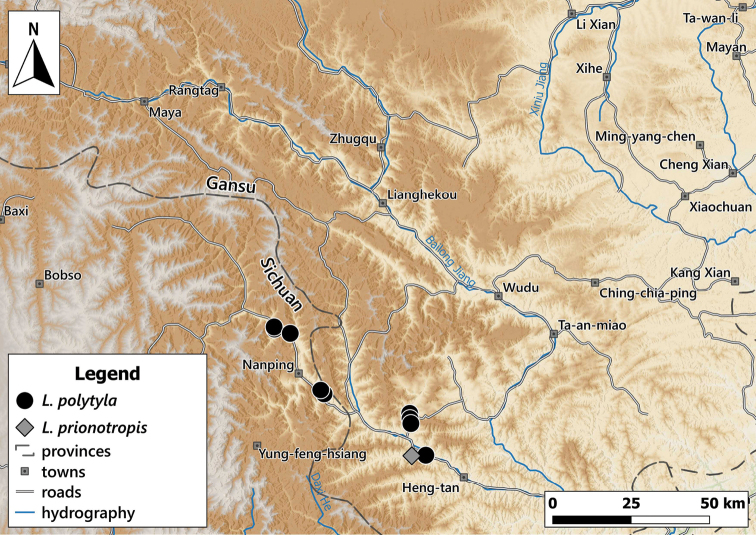
Distribution of *Laeocathaica* species in China (**A** in Fig. 2).

##### New material.

China • 4 shells; Gansu, Longnan Shi, Wenxian, Buziba Xiang, northern edge of Taojiaba Cun, 200 m towards Buziba (locality code: 2016/78); 33°02.706'N, 104°37.157'E; 1200 m a.s.l.; 31 May 2016; A. Hunyadi leg.; HA (Fig. 20B) • 11 shells; Gansu, Longnan Shi, Wenxian, Chengguan Zhen, cemetery hill above the city (locality code: 2016/65); 32°57.026'N, 104°40.527'E; 1090 m a.s.l.; 28 May 2016; A. Hunyadi leg.; HA (Fig. 20C) • 2 shells; Gansu, Longnan Shi, Wenxian, Buziba Xiang, 1 km south of Taojiaba Cun towards Dongyukou Cun (locality code: 2016/79); 33°01.865'N, 104°37.329'E; 1150 m a.s.l.; 31 May 2016; A. Hunyadi leg.; HA • 4 shells; Sichuan, Aba, Jiuzhaigou Xian, Baihe Xiang, Taiping Cun, eastern bank of Baishui He (locality code: 2016/73); 33°18.366'N, 104°09.413'E; 30 May 2016; A. Hunyadi leg.; HA • 7 shells; Sichuan, Aba, Jiuzhaigou Xian, Guoyuan Xiang, Guoyuaner Cun, environment of the bridge (locality code: 2016/76); 33°06.922'N, 104°19.617'E; 1200 m a.s.l.; 30 May 2016; A. Hunyadi leg.; HA • 2 shells; Sichuan, Aba, Jiuzhaigou Xian, Anle Xiang, ca. 1.5 km east of Zhongtianshan Cun towards Jiuzhaigou Shi (locality code: 2016/74); 33°17.279'N, 104°12.702'E; 1445 m a.s.l.; 30 May 2016; A. Hunyadi leg.; HA • 8 shells; Sichuan, Aba, Jiuzhaigou Xian, Baihe Xiang, southern edge of Taiping Cun, rock wall facing north (locality code: 2016/72); 33°18.026'N, 104°09.500'E; 30 May 2016; A. Hunyadi leg.; HA • 3 shells; Gansu, Longnan Shi, Wenxian, Buziba Xiang, southern edge of Buziba Cun, western bank of the river (locality code: 2016/77); 33°03.592'N, 104°37.094'E; 1215 m a.s.l.; 30 May 2016; A. Hunyadi leg.; HA • 2 shells; Sichuan, Jiuzhaigouxian, Guoyuanxiang, 7.7 km from provincial border (locality code: 2011.04.25A); 33°07.616'N, 104°18.927'E; 1258 m a.s.l.; 25 April 2011; Y. Nakahara, K. Okubo & K. Otani leg.; PGB.

##### Distribution.

Known from several precise localities in southern Gansu Province (Fig. 22).

#### ﻿ Laeocathaica potanini

Taxon classificationAnimaliaStylommatophoraBradybaenidae

Möllendorff, 1899

4A727EEA-36DE-51B7-A7D5-CAF95EB86F59

Figure 23A, B

Laeocathaica potanini Möllendorff, 1899: 96–97, pl. 6, fig. 5.
Laeocathaica potanini . – Yen 1939: 149, pl. 15, fig. 38.
Laeocathaica (Laeocathaica) potanini . – Zilch 1968: 174.
Laeocathaica potanini . – Chen and Zhang 2004: 326, fig. 316.

##### Type material.

Gansu: Wenhsien, coll. Möllendorff ex coll. Potanin 251, 587, 734, SMF 9082 (lectotype, Fig. 23A, B) • Same data, SMF 9083/3+1 (paralectotypes, one of them from coll. C. Boettger) • S-Gansu, Hungadan b. Wen-hsien, coll. Möllendorff ex coll. Berezowski, SMF 8960/1.

##### New material.

China • 6 shells; Gansu, Longnan Shi, Wenxian, Chengguan Zhen, cemetery hill above the city (locality code: 2016/65); 32°57.026'N, 104°40.527'E; 1090 m a.s.l.; 28 May 2016; A. Hunyadi leg.; HA • 5 shells; Gansu, Longnan Shi, eastern edge of Wenxian, northern bank of the river (locality code: 2016/66); 32°56.459'N, 104°41.372'E; 28 May 2016; A. Hunyadi leg.; HA.

##### Remarks.

The examined shells are identical to the types.

#### ﻿ Laeocathaica prionotropis

Taxon classificationAnimaliaStylommatophoraBradybaenidae

Möllendorff, 1899

45865CEF-D5BE-5403-90B2-118FC6C921FF

Figures 25 , 26

Laeocathaica prionotropis Möllendorff, 1899: 94–95, pl. 6, figs 1, 1a.
Laeocathaica prionotropis subsp. albocincta Möllendorff, 1899: 95. **new synonym**
Laeocathaica prionotropis prionotropis . – Yen 1939: 149, pl. 15, fig. 34.
Laeocathaica prionotropis albocincta . – Yen 1939: 149, pl. 15, fig. 35.
Laeocathaica (Laeocathaica) prionotropis prionotropis . – Zilch 1968: 175.
Laeocathaica (Laeocathaica) prionotropis albocincta . – Zilch 1968: 175.
Laeocathaica prionotropis . – Chen and Zhang 2004: 320, figs 309–310.

##### Type material.

**Figure 25.**
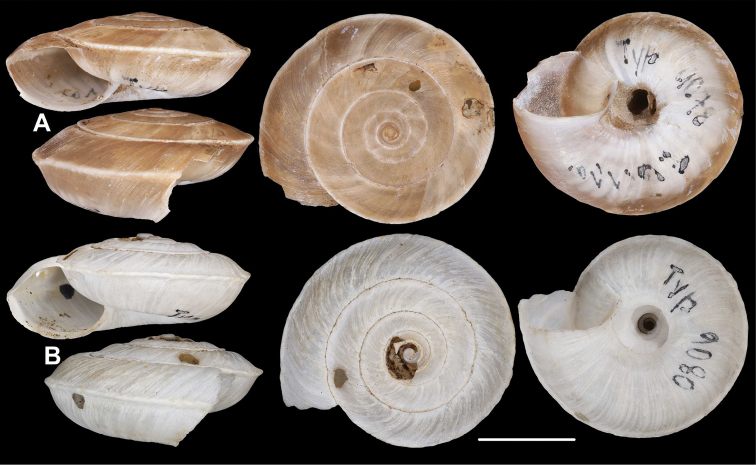
*Laeocathaicaprionotropis***A** lectotype (SMF 9078) **B** lectotype of *Laeocathaicaprionotropisalbocincta* Möllendorff, 1899 (SMF 9080). Scale bar: 10 mm. All photographs: B. Páll-Gergely.

Zw. Yü-lin-guan u. Wen-hsien, coll. Möllendorff ex coll. Potanin 520a, 908a, SMF 9078 (lectotype of *L.prionotropis*, Fig. 25A) • Same data, SMF 9079/3 (paralectotypes) • W. Sy-tshuan, Tung-ho, coll. Möllendorff ex coll. Potanin 312a, SMF 9080 (lectotype of *L.prionotropisalbocincta*, Fig. 25B) • Same data, SMF 9081 (paralectotype of *L.prionotropisalbocincta*).

##### New material.

**Figure 26.**
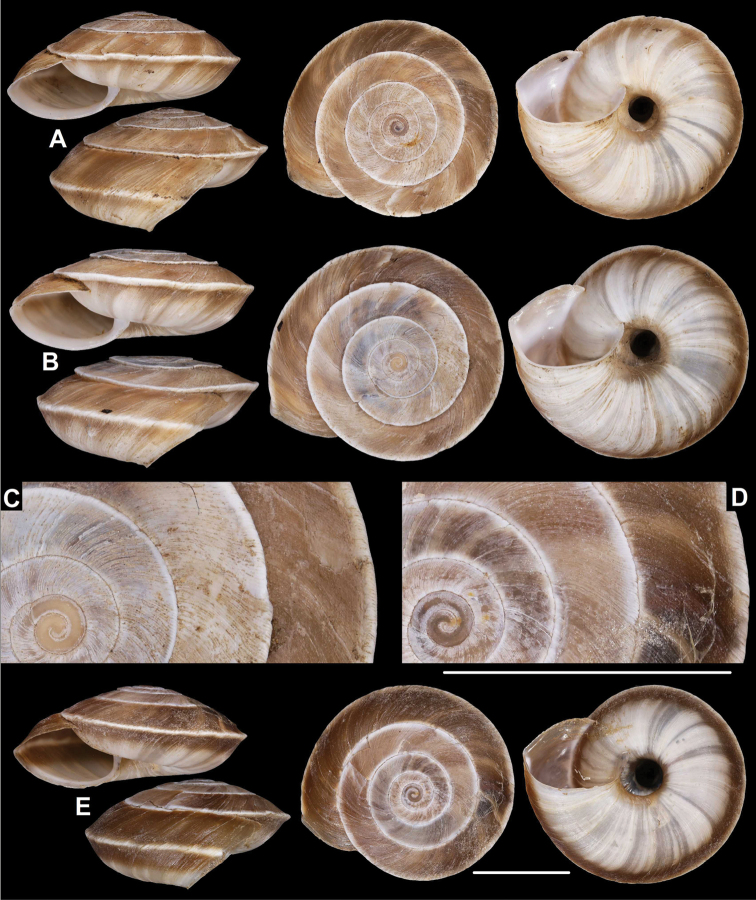
*Laeocathaicaprionotropis***A** 2016/64, specimen1 **B, C** 2016/64, specimen2 **D, E** 2016/63. Scale bars: 10 mm. All photographs: B. Páll-Gergely.

China • 1 shell; Gansu, Longnan Shi, Wenxian, Bikou Zhen, above the hydroelectric power plant, northern side of Bailong He (locality code: 2016/63); 32°45.966'N, 105°13.005'E; 28 May 2016; A. Hunyadi leg.; HA (Fig. 26D, E) • 5 shells; Gansu, Longnan Shi, Wenxian, Chengguan Zhen, next to a museum (locality code: 2016/64); 32°56.471'N, 104°40.379'E; 960–970 m a.s.l.; 28 May 2016; A. Hunyadi leg.; HA (Fig. 26A–C).

##### Distribution.

Known from two sites in Southern Gansu Province (Fig. 24).

##### Remarks.

Laeocathaicaprionotropissubsp.albocincta agrees in size and shell shape with the nominotypical form, and therefore it is here synonymised with *Laeocathaicaprionotropis*.

## ﻿Discussion

Although we list and publish photographs of all *Laeocathaica* species in this work, the taxonomy of this group is still far from being solved. Following previous authors, we classify only sinistral species in *Laeocathaica*. However, it is very probable that the coiling direction has changed multiple times during the evolution of Bradybaeninae inhabiting the arid regions of central China. Furthermore, sinistral species such as *Bradybaenamicromphala* (Möllendorff, 1899) and *B.eris* (Möllendorff, 1899) also inhabit southern Gansu, and are similar to *Laeocathaica* species in most traits except for the narrow umbilicus. Future investigations will probably reveal that the latter two species (and maybe some other similar ones from the region) are relatives of *Laeocathaica* rather than *Bradybaena*.

One of the main outcomes of the present paper is the clarification of some names that have been incorrectly used in the literature and in museum collections because the types were not examined. One such case is Helixchristinaevar.carinifera H. Adams, 1870, which resulted in being a senior synonym of *Laeocathaicasubsimilis* (Deshayes, 1874) after examination of both type species. The other case is that of *L.minwui* sp. nov., which was called *L.christinae* (H. Adams, 1870) in museum collections, because the types of the “real” *L.christinae* had not been examined.

**Figure 27.**
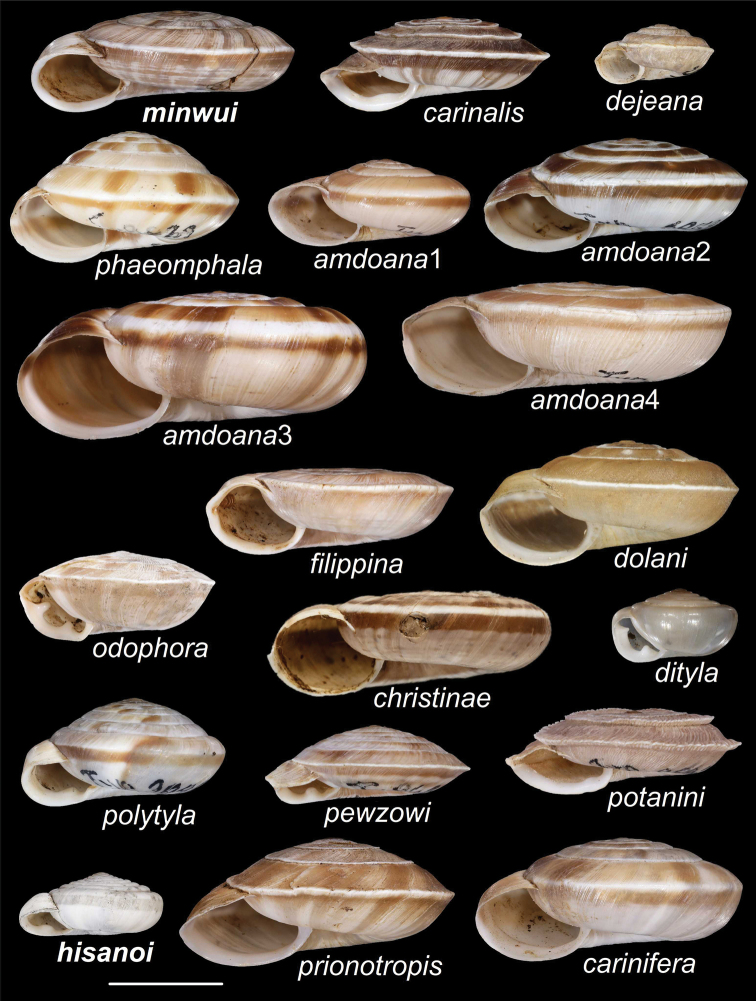
Synoptic view of *Laeocathaica* species. The variability of *L.amdoana* is shown on four examples. Scale bar: 10 mm. Species in bold are described here as new

The other important outcome of the present study is the recognition of continuous dorsal surface variability from flat to domed, the strongly keeled to rounded body whorl, the colouration, and shell size (D: 19–32 mm) across the taxa *Laeocathaicaamdoana*, *L.distinguenda*, *L.tropidorhaphe*, and *L.dangchangensis*.

**Table 4.** Co-occurrence patterns of *Laeocathaica* species with locality codes.

	*L.amdoana*	*L.carinalis*	*L.carinifera*	*L.odophora*	*L.phaeomphala*
***L.carinalis***	2016/64, 2016/79, 2016/82, 2016/83				
***L.carinifera***	2016/67, 2016/68, 2016/70a	2016/70b			
***L.dityla***	2016/88, 2016/95		2016/86		
***L.odophora***	2016/64, 2016/68, 2016/69	2016/64, 2016/70b	2016/68, 2016/70b		
***L.phaeomphala***	2016/64	2016/64		2016/64	
***L.polytyla***	2016/65, 2016/72, 2016/73,2016/74, 2016/76, 2016/77, 2016/78, 2016/79	2016/79	2016/78		
***L.potanini***	2016/65, 2016/66				
***L.prionotropis***	2016/64	2016/64	2016/63	2016/64	2016/64

Table 4 summarises the co-occurrence patterns between *Laeocathaica* species, showing which species pairs are reproductively isolated, true biological species, and Fig. 27 shows all *Laeocathaica* species.

## Supplementary Material

XML Treatment for Cathaica

XML Treatment for Cathaica (Cathaica) bizonalis

XML Treatment for Laeocathaica

XML Treatment for Laeocathaica amdoana

XML Treatment for Laeocathaica carinalis

XML Treatment for Laeocathaica carinifera

XML Treatment for Laeocathaica christinae

XML Treatment for Laeocathaica dejeana

XML Treatment for Laeocathaica dityla

XML Treatment for Laeocathaica dolani

XML Treatment for Laeocathaica filippina

XML Treatment for Laeocathaica hisanoi

XML Treatment for Laeocathaica leucorhaphe

XML Treatment for Laeocathaica minwui

XML Treatment for Laeocathaica odophora

XML Treatment for Laeocathaica pewzowi

XML Treatment for Laeocathaica phaeomphala

XML Treatment for Laeocathaica polytyla

XML Treatment for Laeocathaica potanini

XML Treatment for Laeocathaica prionotropis
